# Recognition of mixed-sequence double-stranded DNA regions using chimeric Invader/LNA probes†

**DOI:** 10.1039/d4ob01403k

**Published:** 2024-10-08

**Authors:** Michaela E. Everly, Raymond G. Emehiser, Patrick J. Hrdlicka

**Affiliations:** a Department of Chemistry, University of Idaho Moscow Idaho 83844-2343 USA hrdlicka@uidaho.edu

## Abstract

Development of robust oligonucleotide-based probe technologies, capable of recognizing specific regions of double-stranded DNA (dsDNA) targets, continues to attract considerable attention due to the promise of tools for modulation of gene expression, diagnostic agents, and new modalities against genetic diseases. Our laboratory pursues the development of various strand-invading probes. These include Invader probes, *i.e.*, double-stranded oligonucleotide probes with one or more +1 interstrand zipper arrangements of intercalator-functionalized nucleotides like 2′-*O*-(pyren-1-yl)methyl-RNA monomers, and chimeric Invader/γPNA probes, *i.e.*, heteroduplex probes between individual Invader strands and complementary γPNA strands. Here we report on the biophysical properties and dsDNA-recognition characteristics of a new class of chimeric probes—chimeric Invader/LNA probes—which are comprised of densely modified Invader strands and fully modified complementary LNA strands. The chimeric Invader/LNA probes form labile and distorted heteroduplexes, due to an apparent incompatibility between intercalating pyrene moieties and LNA strands. In contrast, the individual Invader and LNA strands form very stable duplexes with complementary DNA, which provides the driving force for near-stoichiometric recognition of model double-stranded DNA targets with single base-pair accuracy. The distinctive properties of chimeric Invader/LNA probes unlock exciting possibilities in molecular biology, and diagnostic and therapeutic fields.

## Abstract 2

Heteroduplexes comprised of densely intercalator-modified oligodeoxyribonucleotides and LNAs (locked nucleic acids) enable highly specific mixed-sequence recognition of double-stranded DNA regions.

## Graphical abstract

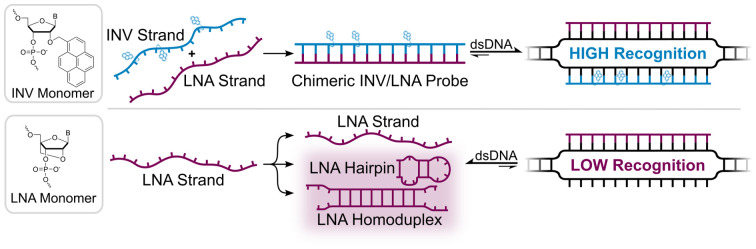

## Introduction

While approximately twenty RNA-targeting antisense oligonucleotides (ONs), splice-switching ONs, and small interfering RNAs (siRNAs) have received regulatory approval in recent years as therapeutics against genetic diseases,^1^ no DNA-targeting ONs have entered the clinic. This reflects the additional pharmacodynamic and pharmacokinetic challenges associated with DNA-targeting ONs that include the nuclear localization and highly condensed nature of chromosomal DNA, which presents few nucleotide-specific signatures for exogenous ligands to recognize. Development of robust, ON-based DNA-targeting probe technologies as alternatives to the popular, protein-based CRISPR-Cas9 (clustered regularly interspaced short palindromic repeats (CRISPR)-associated protein 9) constructs—which suffer from immunogenicity of CRISPR components, delivery challenges, low specificity and off-target effects^2^—is highly desirable given their potential applications as diagnostic agents, modulators of gene expression, and therapeutic modalities against genetic diseases.

Major efforts have been devoted to the development of DNA-targeting probe technologies in recent decades. Pioneering approaches include pyrrole-imidazole polyamides^3,4^ and triplex-forming ONs^5,6^ and peptide nucleic acids (PNAs),^6,7^ which access nucleotide-specific features from the duplex grooves. However, triplex formation in the major groove is normally restricted to regions with extended polypurine tracts, whereas polyamides are generally only directed to short dsDNA regions as binding- and shape-complementarity in the minor groove is compromised when longer regions are targeted. Although polyamides can be linked to target longer dsDNA regions,^8^ and strategies reducing the polypurine requirement for triplex formation have been developed,^6,9–11^ this increases the synthetic complexity of the probes without fully addressing the pharmacodynamic and pharmacokinetic challenges.

The limitations of groove-binding approaches has prompted the development of strand-invading probe technologies, *i.e.*, probes capable of breaking the existing Watson-Crick base-pairs of dsDNA regions to form new, more stable base-pairs between probe strands and complementary DNA (cDNA) regions. Examples include variously modified single-stranded PNAs^12–17^ and locked nucleic acids (LNAs),^18–21^ as well as double-stranded probes like pseudo-complementary PNAs^22–28^ and related approaches.^29^ A key advantage of strand-invading probes is that mixed-sequence dsDNA regions can be targeted. However, single-stranded probes with high cDNA affinity often tend to self-associate, which may compromise target binding.^27^ Double-stranded probes, in turn, must be engineered to denature easily, whilst maintaining high cDNA affinity.

**Fig. 1:**
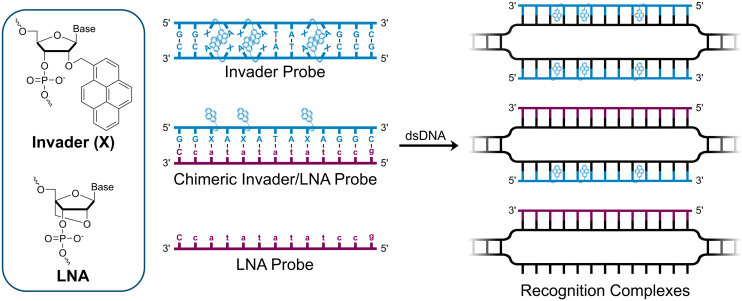
Structures of monomers and illustrations of dsDNA-recognition mechanisms using Invader probes, chimeric Invader/LNA probes, and single-stranded LNAs. DNA monomers are denoted in upper case letters, whereas LNA monomers are denoted in lower case letters (“*c*” = 5-methyl-cytosin-1-yl LNA monomer).

We have pursued the development of strand-invading probes called Invader probes.^30–32^ These are double-stranded oligonucleotide probes featuring one or more +1 interstrand zipper arrangements^33^ of intercalator-functionalized nucleotides like 2′-*O*-(pyren-1-yl)methyl-RNA (Fig. 1). This specific arrangement of covalently linked intercalators (termed *energetic hotspot*) forces the intercalators between the π-stacks of neighboring base-pairs resulting in violation of the nearest neighbor exclusion principle^34,35^ (NNEP) and the formation of an unwound and destabilized probe duplex, as the local intercalator density is too high.^36^ The two probe strands, in turn, display high affinity toward cDNA regions as duplex formation results in strongly stabilizing stacking interactions between the intercalator and flanking base-pairs (local intercalator density is low and does not violate the NNEP) (Fig. 1). The stability differences between the two double-stranded probe/cDNA segments, the double-stranded Invader probe, and the dsDNA target region provide the driving force for mixed-sequence dsDNA-recognition *via* a double-duplex invasion process (Fig. 1).^30–32,37^

We have previously used Invader probes for mixed-sequence recognition of (i) DNA fragments from specific foodborne pathogens using a sandwich assay,^38^ (ii) telomeric DNA of individual chromosomes in metaphasic spreads,^39^ and (iii) target regions on bovine Y-chromosomes in interphase and metaphase nuclei under non-denaturing conditions,^32,40^ which highlights the potential applications of DNA-targeting Invader probes.

In addition to improving the dsDNA-recognition characteristics of Invader probes through optimization of the intercalator-functionalized monomer and probe architecture,^41–43^ we recently introduced chimeric probes between individual Invader strands and complementary miniPEG-γPNA (^MP^γPNA), serine-γPNA (^Ser^γPNA), or LNA-modified oligodeoxyribonucleotides (ODNs) as an extension of our original strategy.^44,45^ These efforts were inspired by prior studies on DNA-targeting heteroduplex probes between intercalator-modified ONs and complementary RNA, PNA, or LNA strands.^46–48^ These approaches rely on the observation that intercalators are typically accommodated poorly in PNA/DNA duplexes and *A*-type (RNA-like) duplexes,^49,50^ but well-tolerated in *B*-type (DNA-like) duplexes. As a result, the chimeric heteroduplex probes are more labile than the corresponding duplexes between individual probes strands and cDNA. Along a similar vein, chimeric Invader/^MP^γPNA and Invader/^Ser^γPNA probes were found to be energetically activated for dsDNA-recognition, resulting in more efficient and specific recognition than the corresponding single-stranded ^MP^γPNA and ^Ser^γPNA.^44,45^ This was attributed to the Invader strand's ability to bind and sequester formation of secondary γPNA structures that are refractory to dsDNA-recognition. In contrast, chimeric probe duplexes comprised of individual Invader strands and partially LNA-modified ODNs were only weakly activated for dsDNA-recognition, presumably because the LNA modification levels were insufficient to effectively impede intercalation of the pyrene moieties.^44^

Here, we report on the biophysical properties and dsDNA-recognition characteristics of chimeric probes comprised of Invader strands and fully modified^51^ LNA strands. The constructs were expected to be more strongly activated for mixed-sequence dsDNA-recognition for at least two reasons. First, the high LNA content, which is known to increasingly tune DNA duplex structures towards more *A*-type geometries,^52^ was expected to disfavor pyrene intercalation and thus yield more labile probes. Secondly, both Invader^32,40^ and LNA^53^ strands are known to display high cDNA affinity, which we expected would generate a more prominent driving force for mixed-sequence dsDNA-recognition.

## Results and discussion

### Design and synthesis of probes

Individual Invader probe strands—featuring two, three or four 2′-*O*-(pyren-1-yl)methyl-uridine monomers—were available from prior studies^32,39^ whereas complementary, fully modified^51^ LNA strands were synthesized and purified following established protocols (see Experimental section). Access to these ONs enabled us to evaluate fourteen probes (Table 1), *i.e.*, eight chimeric Invader/LNA probes (χ1–χ8) and four conventional Invader probes (INV1:INV2–INV7:INV8) with two, three, or four 2′-*O*-(pyren-1-yl)methyluridine monomers per Invader strand (subsequently referred to as 2X-, 3X-, and 4X-modified chimeric or Invader probes), as well as two single-stranded LNA controls (LNA1 and LNA2). These probes were designed to target a 13-mer partially self-complementary dsDNA region that we have previously used as a model target to evaluate various Invader and chimeric probes.^32,39^

**Table 1:** LNA, Invader, and chimeric probes studied herein; *T*_m_s of probe duplexes and duplexes between individual probe strands and cDNA, and TA values^a^

		*T*_m_ [Δ*T*_m_]^b^ (°C)			
Name	Probe sequence	Probe	Upper strand *vs.* cDNA	Lower strand *vs.* cDNA	TA (°C)
χ1	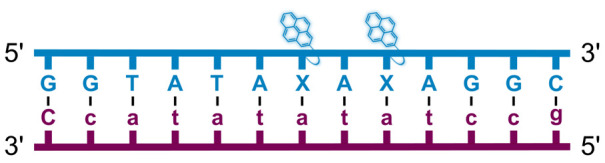	44.0	54.5	68.5	41.5
		[+6.5]	[+17.0]	[+31.0]	
χ2	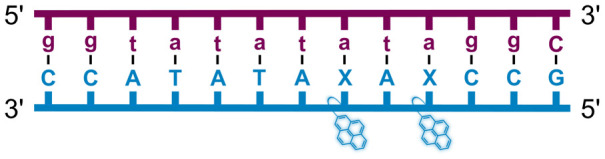	49.0	71.0	54.5	39.0
		[+11.5]	[+33.5]	[+17.0]	
χ3	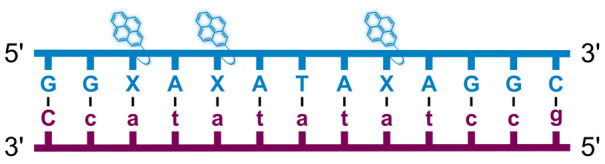	33.0	61.0	68.5	59.0
		[−4.5]	[+23.5]	[+31.0]	
χ4	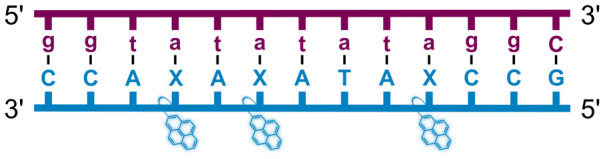	31.5	71.0	63.0	65.0
		[−6.0]	[+33.5]	[+25.5]	
χ5	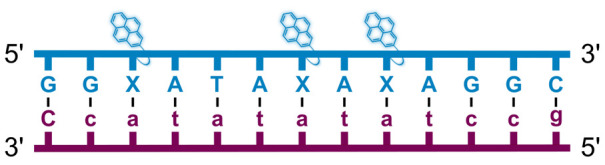	∼32.0^c^	61.5	68.5	∼60.5
		[−5.5]	[+24.0]	[+31.0]	
χ6	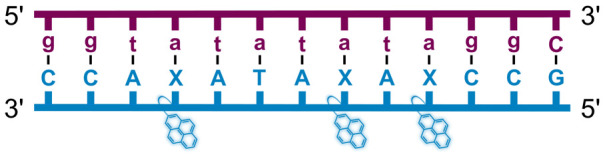	∼32.0^c^	71.0	62.5	∼64.0
		[−5.5]	[+33.5]	[+25.0]	
χ7	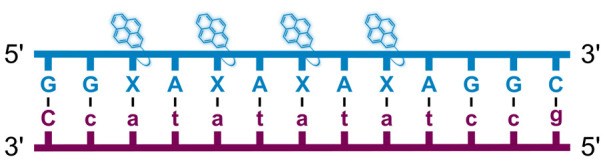	∼33.5^c^	65.5	68.5	∼63.0
		[−4.0]	[+28.0]	[+31.0]	
χ8	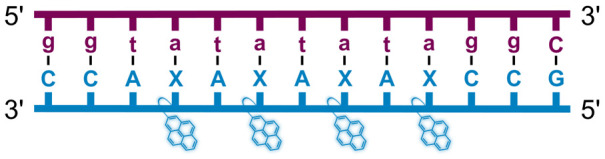	∼41.5^c^	71.0	67.5	∼59.5
		[+4.0]	[+33.5]	[+30.0]	
INV1	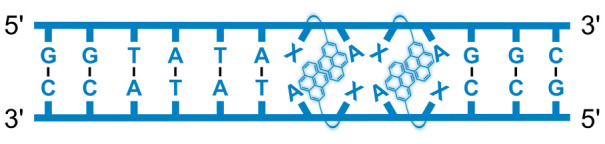	47.5	54.5	54.5	24.0
INV2		[+10.0]	[+17.0]	[+17.0]	
INV3	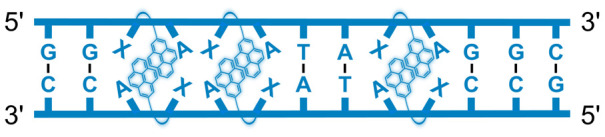	51.0	61.0	63.0	35.5
INV4		[+13.5]	[+23.5]	[+25.5]	
INV5	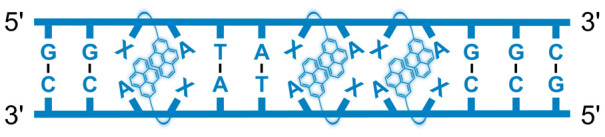	52.0	61.5	62.5	34.5
INV6		[+14.5]	[+24.0]	[+25.0]	
INV7	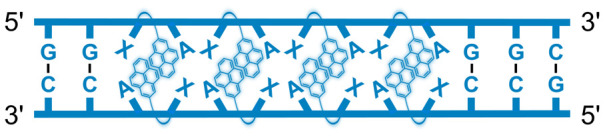	50.0	65.5	67.5	45.5
INV8		[+12.5]	[+28.0]	[+30.0]	
LNA1	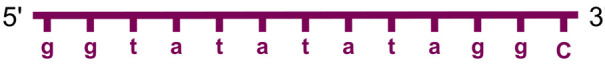	>80.0^d^	71.0	—	—
		[>+42.5]	[+33.5]		
LNA2	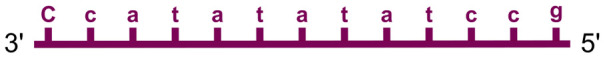	>80.0^d^	—	68.5	—
		[>+42.5]		[+31.0]	

a LNA strands shown in purple. Invader strands, featuring 2′-*O*-(pyren-1-yl)methyluridine X monomers, shown in blue. Chimeric probes are comprised of the following strands: χ1 (INV1 : LNA2), χ2 (INV2 : LNA1), χ3 (INV3 : LNA2), χ4 (INV4 : LNA1), χ5 (INV5 : LNA2), χ6 (INV6 : LNA1), χ7 (INV7 : LNA2), and χ8 (INV8 : LNA1). Structures of modifications are shown in Fig. 1. Δ*T*_m_ = change in *T*_m_ relative to the unmodified DNA duplex, *T*_m_ (5′-GGTATATATAGGC:3′-CCATATATATCCG) = 37.5 °C. Thermal denaturation curves (Fig. S2†) were recorded in a medium salt phosphate buffer ([Na^+^] = 110 mM, [Cl^−^] = 100 mM, pH 7.0 (NaH_2_PO_4_/Na_2_HPO_4_), [EDTA] = 0.2 mM) using 1.0 μM of each strand. “—” = not applicable. TA is defined in the main text. *T*_m_s and TAs for Invader probes have been previously reported in ref. 32 and 39.

b *T*_m_s for chimeric probes and LNA/cDNA duplexes were obtained from differential thermal denaturation curves (Fig. S4†).

c Irregular profiles and broad transitions were observed (Fig. S2 and S4†).

d For additional discussion of *T*_m_ values for LNA1 and LNA2, see ESI.†

### Thermal denaturation temperatures and dsDNA-targeting potential

Thermal denaturation temperatures (*T*_m_s) were determined for the double-stranded probes (*i.e.*, chimeric Invader/LNA and conventional Invader probes) and duplexes between individual probe strands and cDNA (Table 1). Thermal denaturation profiles were also recorded for single-stranded LNAs to study the potential formation of secondary structures.

*T*_m_s were used to calculate the thermal advantage, a term that we have used to estimate the driving force for recognition of complementary dsDNA targets by double-stranded probes,^32^ and which we define as TA = *T*_m_ (upper strand *vs.* cDNA) + *T*_m_ (lower strand *vs.* cDNA) − *T*_m_ (probe duplex) − *T*_m_ (dsDNA). More positive values indicate a more prominent driving force for dsDNA-recognition.

As reported previously,^32,39^ Invader probes with two, three, or four energetic hotspots are more labile than the corresponding duplexes between individual Invader strands and cDNA (*T*_m_s = 47.5–52.0 °C *vs. T*_m_s = 54.5–67.5 °C, respectively), but more stable than the unmodified DNA duplex (*T*_m_ = 37.5 °C). As expected, duplexes between LNA strands and cDNA were found to be exceptionally stable (*T*_m_s = 68.5–71.0 °C).

The raw thermal denaturation profiles of the chimeric Invader/LNA probes—like those of chimeric Invader/^Ser^γPNA probes^45^—were irregular (Fig. S2†), indicating the formation of distorted duplexes which rendered accurate determination of *T*_m_ values challenging. In addition, the LNA strands were prone to formation of secondary structures (*vide infra*). We therefore determined *T*_m_ values for the chimeric Invader/LNA probes from differential thermal denaturation curves, in which the denaturation profiles for single-stranded LNAs were subtracted from the denaturation profiles of the chimeric probes (Fig. S4—for a full discussion, see ESI†). The chimeric probes displayed *T*_m_ values in the 30–50 °C range (Table 1), with the more highly modified chimeric probes being more labile than the corresponding Invader probes (*e.g.*, compare *T*_m_s of χ7 and χ8 with INV7:INV8). The labile nature of the chimeric probes, coupled with the very high cDNA affinity of the LNA strands, resulted in more positive TA values than for the corresponding Invader probes. Thus, TA values were found to range between 24.0–45.5 °C for Invader probes and 39.0–65.0 °C for chimeric Invader/LNA probes, with more prominent TA values observed for 3X- and 4X-modified chimeric probes.

Low- and high-temperature transitions were observed for the single-stranded LNAs, indicating formation of stable secondary structures in this partially self-complementary sequence context (see ESI† for a detailed discussion). The formation of stable secondary structures, a common feature of single-stranded probes featuring affinity-enhancing modifications,^27,28,39,44^ should be expected to reduce the dsDNA-recognition capacity of the single-stranded LNAs.

### Recognition of mixed-sequence dsDNA model targets: design and initial screen

**Fig. 2:**
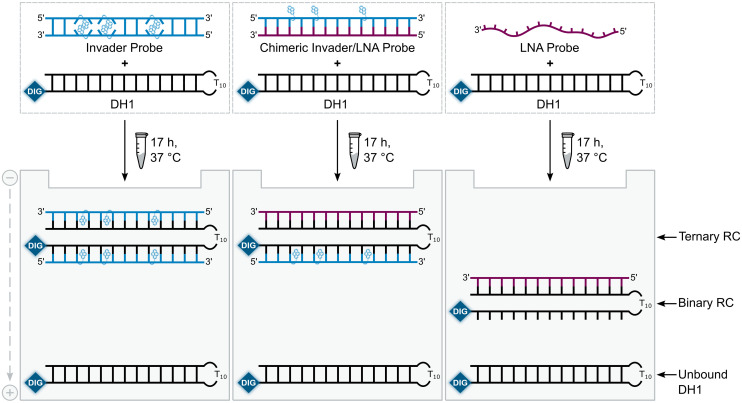
Illustration of the EMSA used to evaluate dsDNA-recognition of probes used herein. RC = recognition complex.

A subset of chimeric Invader/LNA probes were evaluated alongside conventional Invader probes, and single-stranded LNA and Invader strands for their ability to recognize a digoxigenin (DIG)-labeled DNA hairpin (DH1) in an electrophoretic mobility shift assay (EMSA) (Fig. 2). This model target is comprised of a 13-mer double-stranded target region that is complementary to the probes and linked on one end *via* a *T*_10_ loop, resulting in a high-melting hairpin (*T*_m_ = 58.5 °C, Table S3†). Successful recognition of DH1 is expected to result in the formation of binary or ternary complexes (with single-stranded or double-stranded probes, respectively), with reduced electrophoretic mobility on non-denaturing polyacrylamide (nd-PAGE) gels relative to DH1 (Fig. 2).

**Fig. 3:**
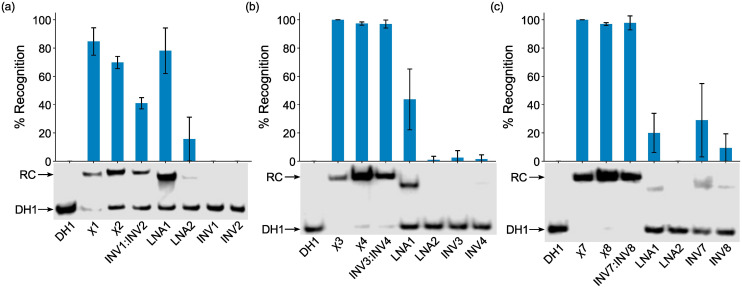
Representative electrophoretograms for recognition experiments between model dsDNA target DH1 and (a) 25-fold, (b) 5-fold, or (c) 2-fold molar excess of various probes (left lanes contain DH1 only). Histograms show averaged results from at least three experiments with error bars representing standard deviation. Data are tabulated in Table 2. RC = recognition complex. DIG-labeled DH1 (50 nM, 5′-GGTATATATAGGC-T_10_-GCCTATATATACC-3′) was incubated with the specified probe in HEPES buffer (50 mM HEPES, 100 mM NaCl, 5 mM MgCl_2_, pH 7.2, 10% sucrose, 1.44 mM spermine tetrahydrochloride) at 37 °C for ∼17 h and subsequently resolved on 20% nd-PAGE gels.

The probes were initially screened against DH1 using a 25-fold, 5-fold, and 2-fold molar probe excess for the 2X-, 3X-, and 4X-modified probes, respectively, and an incubation temperature of 37 °C. Recognition of DH1 was observed with all chimeric and Invader probes (Fig. 3). The 2X-modified chimeric probes χ1 and χ2 resulted in ∼85% and ∼70% recognition, respectively, whilst recognition with the corresponding Invader probe was less efficient (INV1:INV2, ∼40%, Fig. 3a). Remarkably, the 3X- and 4X-modified chimeric and Invader probes resulted in near-complete recognition of DH1 (Fig. 3b and c).

As expected from our prior studies,^32^ individual Invader strands resulted in little-to-no recognition of DH1 at the concentrations used, demonstrating that both strands of an Invader probe typically are needed to ensure efficient dsDNA-recognition. Similarly, single-stranded LNA2 also only resulted in no or minimal recognition of DH1. Surprisingly, given the proclivity of LNA strands to form secondary structures in the conditions of the thermal denaturation experiments (Fig. S3†), LNA1 resulted in substantial recognition of DH1 (∼20%, ∼45%, and ∼80% when used at 2-fold, 5-fold, and 25-fold molar excess, respectively, Fig. 3). While these observations are not fully understood, we (a) note that LNA1 displays slightly greater cDNA affinity than LNA2 (*T*_m_s = 71.0 °C *vs.* 68.5 °C, Table 1) and (b) speculate that the different incubation conditions of the EMSA *vis-à-vis* the thermal denaturation experiments preclude the formation of secondary structures for LNA1 but not for LNA2. Similar observations were previously made for two corresponding ^MP^γPNAs in this sequence context.^39^

### Recognition of model mixed-sequence dsDNA targets: dose–response and binding specificity

**Fig. 4:**
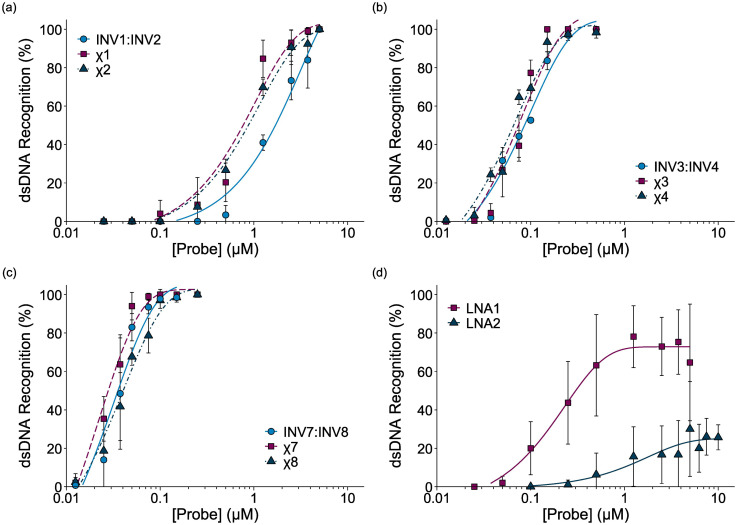
Dose-response curves for recognition of DH1 by (a) 2X-, (b) 3X-, or (c) 4X-modified probe duplexes, and (d) individual LNA strands. For experimental conditions, see Fig. 3. For representative gel electrophoretograms, see Figs. S6 and S8.†

Dose-response experiments were conducted for certain chimeric Invader/LNA probes, Invader probes, and single-stranded LNAs to determine *C*_50_ values, *i.e.*, the probe concentrations resulting in 50% recognition of DH1 (Fig. 4 and Fig. S6–S8†). Lower *C*_50_ values were observed with increasing number of 2′-*O*-(pyren-1-yl)methyluridine incorporations (Table 2). The 2X-modified chimeric probes displayed *C*_50_ values of 0.77–0.87 μM, while the *C*_50_ value for the corresponding Invader probe was roughly two-fold higher. Remarkably, the *C*_50_ values for the 3X-modified chimeric and Invader probes were an order of magnitude lower (65–85 nM), while the 4X-modified probes displayed near-stoichiometric recognition of DH1 (30–40 nM).

**Table 2:** C_50_ values and percent recognition of DH1 by chimeric Invader/LNA probes, Invader probes, and single-stranded LNAs.^a^

Probe	C_50_ (nM)	Rec_2X_ (%)	Rec_5X_ (%)	Rec_25X_ (%)
χ1	770	4 ± 7	9 ± 14	85 ± 10
χ2	870	0 ± 0	8 ± 7	70 ± 4
INV1:INV2	1700	0 ± 0	0 ± 0	41 ± 4
χ3	74	77 ± 7	100 ± 0	n.d.
χ4	66	69 ± 7	97 ± 1	n.d.
INV3:INV4	86	52 ± 1	97 ± 3	n.d.
χ7	28	100 ± 0	100 ± 0	n.d.
χ8	40	97 ± 1	100 ± 0	n.d.
INV7:INV8	36	98 ± 2	n.d.	n.d.
LNA1	300	20 ± 14	44 ± 22	78 ± 16
LNA2	>10 000	0 ± 0	1 ± 2	16 ± 16

a Calculated from dose-response curves shown in Fig. 4. Rec_2X_, Rec_5X_, and Rec_25X_ = percent of DH1 recognition when using 2-fold, 5-fold, and 25-fold molar probe excess, respectively; “±” = standard deviation from at least three trials. n.d. = not determined. Data for χ5 are shown in Fig. S7.†

Interestingly, the single-stranded LNA1 resulted in more efficient recognition of DH1 than the 2X-modified chimeric and conventional Invader probes (*C*_50_ = 0.30 μM), whereas LNA2 did not result in substantial recognition of DH1 (*C*_50_ > 10 μM). It should be noted that the extent of DH1-recognition by LNA1 varied considerably between experiments (note the error bars in Fig. 4d), presumably due to the formation of dynamic secondary structures. This indicates that certain, but not all, LNAs can target mixed-sequence DNA targets with moderate efficiency.

**Fig. 5:**
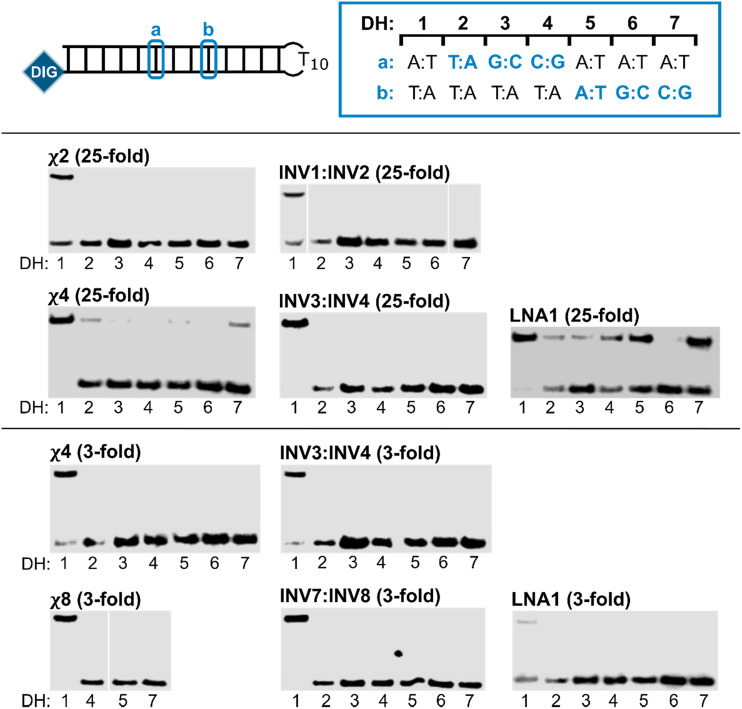
Binding specificity of chimeric Invader/LNA probes, Invader probes, and single-stranded LNAs. Illustration of DH1–DH7 (upper panel). Representative electrophoretograms from experiments in which DH1–DH7 were incubated with the indicated probes at 25-fold (middle panel) or 3-fold molar excess (lower panel). For sequences and *T*_m_s of DH1–DH7, see Table S3.† Incubation conditions are as described in Fig. 3. The electrophoretogram for INV1:INV2 is a composite of two gels, while the electrophoretogram for χ8 is from one gel, with an irrelevant lane excised.^54^

Next, the binding specificities of chimeric probes constructed using LNA1 (*i.e.*, χ2, χ4, and χ8) and the corresponding Invader probes were evaluated alongside LNA1. DIG-labeled DNA hairpins (DH2–DH7), with fully base-paired stems differing in sequence at either position 6 or 9 (a or b, respectively, Fig. 5—upper panel) relative to the probes, were incubated with a 3-fold or 25-fold molar probe excess.

The chimeric Invader/LNA probes display excellent binding specificity, as evidenced by the (near-)complete absence of recognition bands when the probes are incubated with DH2–DH7 at conditions that result in substantial or complete recognition of DH1 (Fig. 5). Thus, no recognition of DH2–DH7 is observed when a 25-fold excess of χ2 (Fig. 5—middle panel) or three-fold excess of χ4 (Fig. 5—lower panel) is used. Minor recognition of DH2 and DH7 is observed when a 25-fold excess of χ4 is used, while no more than trace recognition of the remaining non-target hairpins is observed (Fig. 5—middle panel). Remarkably, high-affinity probe χ8 displays complete discrimination of non-target hairpins when used at 3-fold excess (Fig. 5—lower panel).^54^ Similarly, the corresponding Invader probes display complete discrimination of non-target DNA hairpins DH2–DH7, as evidenced by the absence of recognition bands. In contrast, the single-stranded LNA1 displays mediocre binding specificity when used at 25-fold excess (Fig. 5—middle panel), whereas hardly any recognition of matched or mismatched targets is observed when LNA1 is used at 3-fold excess (Fig. 5—lower panel).

Presumably, the excellent binding specificity of the double-stranded chimeric and Invader probes is due to stringency clamping effects that are often observed with structured probes.^55^ Thus, the double-stranded Invader probes or chimeric Invader/LNA probes are less stable than the ternary recognition complexes formed with the correct DNA hairpin target, but more stable than the complexes featuring two destabilized mismatched duplexes that would form with non-target hairpins. The double-stranded probes act as stringency clamps by interfering with non-target binding and widening the window of conditions for affinity and specificity. In contrast, only one mismatched duplex needs to form with single-stranded high-affinity probes like LNA1. Accordingly, these results highlight one of the key advantages of double-stranded probes for recognition of mixed-sequence dsDNA target regions.

## Conclusion

Double-stranded chimeric probes, comprised of densely modified Invader strands (*i.e.*, ODNs modified with ∼30% interspersed 2′-*O*-(pyren-1-yl)methyl-RNA monomers) and complementary fully modified LNA strands, enable efficient and highly specific recognition of model mixed-sequence DNA targets. The driving force for DNA-recognition is due to (a) the labile nature of the chimeric heteroduplexes, and (b) the high affinity of Invader and LNA strands towards cDNA. Unlike high-affinity single-stranded probes—*e.g.*, various single-stranded PNAs^39,45^ or, as presented herein, LNAs—which often suffer from unintended formation of stable secondary structures that may interfere with DNA-recognition, the chimeric Invader/LNA probes are engineered to form labile and distorted heteroduplexes that facilitate DNA-recognition. Thus, chimeric Invader/LNA probes are a valuable addition to the growing class of double-stranded DNA-targeting probes like pseudo-complementary PNA,^23–28^ conventional Invader probes,^30–32,37^ and other heteroduplex probes,^44–48^ that enable mixed-sequence recognition of DNA duplex regions. Optimizations and life science applications of chimeric Invader/LNA probes are contemplated and will be reported in due course.

## Experimental section

### Synthesis and purification of probe strands

All Invader strands used herein were available from prior studies.^32,39^ LNA-modified ODNs were synthesized on an automated DNA synthesizer using long chain alkyl amine controlled pore glass (LCAA-CPG) solid support with a pore size of 500 Å. LNAs were deprotected and cleaved from the solid support *via* treatment with 32% ammonia (55 °C, 17 h). The ammonia solution was then evaporated off, and the crude LNAs reconstituted in water, detritylated (5% aq. CF_3_COOH, RT) and purified using TOP-DNA 150 mg tube oligonucleotide cartridges (Agilent) (50:50 MeCN:H_2_O, v/v). The purity and identity of the synthesized LNA-modified strands was verified using analytical HPLC (XTerra MS C_18_ column: 0.05 M triethyl ammonium acetate and acetonitrile gradient; >90% and >95% purity for LNA1 and LNA2, respectively, Fig. S1†) and LC-ESI-MS analysis (Waters Acquity C_18_ column; triethyl ammonium acetate and acetonitrile gradient) recorded on a quadrupole time-of-flight (Q-TOF) mass spectrometer. The raw signals were deconvoluted using the Max Ent software provided with the spectrometer to obtain molecular ion peaks (Table S1 and Fig. S1†).

### Thermal denaturation and UV-Vis experiments

Concentrations of Invader strands were estimated using the following DNA extinction coefficients (OD_260_/μmol): G (12.01), A (15.20), T (8.40), C (7.05), and pyrene (22.4).^56^ Concentrations of the fully modified LNA strands were estimated using the following RNA extinction coefficients (OD_260_/μmol): G (13.7), A (15.4), T (10.0), and ^5-Me^C (9.0). Thermal denaturation temperatures of duplexes (1.0 μM final concentration of each strand) were determined on a Cary 100 UV/VIS spectrophotometer equipped with a 12-cell Peltier temperature controller and measured as the maximum of the first derivative of thermal denaturation curves (*A*_260_*vs. T*) recorded in medium salt buffer unless otherwise specified ([Na^+^] = 110 mM, [Cl^−^] = 100 mM, pH 7.0 (NaH_2_PO_4_/Na_2_HPO_4_), [EDTA] = 0.2 mM). Strands were mixed in quartz optical cells with a path length of 1.0 cm and annealed by heating to 90 °C (2 min) followed by cooling to the starting temperature of the experiment. The temperature of the denaturation experiments ranged from at least 15 °C below the *T*_m_ to at least 15 °C above the *T*_m_ (although not above 95 °C). A temperature ramp of 0.5 °C min^−1^ was used in all experiments. Reported *T*_m_s are averages of at least two experiments within ± 1.0 °C. *T*_m_s for chimeric probes and LNA/cDNA duplexes were determined from differential thermal denaturation curves to eliminate the impact of LNA-based secondary structure denaturation.

Absorption spectra (range 200–600 nm) were recorded at 10 °C using the same samples (*i.e.*, each strand used at 1.0 μM in *T*_m_ buffer) and instrumentation as in the thermal denaturation experiments (see Fig. S5, Table S2 and ESI† for discussion).

### Electrophoretic mobility shift assays

DNA hairpins were obtained from commercial sources and used without further purification. Hairpins were 3′-labelled with digoxigenin (DIG) using the 3′-end labeling procedure provided with the 2^nd^ Generation DIG Oligonucleotide 3′-End Labeling Kit (Roche). Briefly, 11-digoxigenin-ddUTP was incorporated at the 3′-end of the hairpin (100 pmol) using a recombinant terminal transferase. The reaction was quenched through addition of EDTA (0.05 M), and the mixture diluted to 100 nM and used without further processing.

Solutions of chimeric, Invader, and LNA probes (concentrations as specified) were incubated with the corresponding DIG-labeled DNA hairpin (final concentration 50 nM) in HEPES buffer (50 mM HEPES, 100 mM NaCl, 5 mM MgCl_2_, pH 7.2, 10% sucrose, 1.44 mM spermine tetrahydrochloride) at 37 °C for the specified time. Following incubation, loading dye (6X) was added and the mixtures were loaded onto 20% non-denaturing TBE-PAGE slabs (45 mM tris-borate, 1 mM EDTA; acrylamide : bisacrylamide (19:1)). Incubation mixtures were resolved *via* electrophoresis, which was performed using constant voltage (∼70 V) at ∼4 °C for ∼4 h.

Bands were subsequently blotted onto positively charged nylon membranes (∼100 V, ∼30 min, ∼4 °C) and cross-linked through exposure to UV light (254 nm, 5 × 15 W bulbs, 5 min). Membranes were then incubated with anti-digoxigenin-alkaline phosphatase *F*_ab_ fragments as recommended by the manufacturer and transferred to a hybridization jacket. Membranes were then incubated with the chemiluminescence substrate (CSPD) for 10 min at 37 °C followed by ∼3 h at room temperature whilst being shielded from light. Chemiluminescence of the formed product was captured and quantified (as the intensity ratios between the bands corresponding to the recognition complexes and unbound hairpin) using the C-DiGit® Blot Scanner (LI-COR) and accompanying software (Image Studio). An average of at least three independent experiments for recognition and dose-response assays, and at least two independent experiments for binding specificity assays (unless otherwise noted), is reported along with standard deviations (±). The shown electrophoretograms are in some instances composite images of lanes from different runs (indicated in the respective figure legends).

The Levenberg-Marquardt nonlinear least-squares algorithm from the MINPACK library in R was used to fit data points from dose-response experiments to the following equation: *y* = *C* + *A* (1 − *e*^−*kt*^) where *C*, *A* and *k* are fitting constants.^57^ The resulting equation was used to calculate *C*_50_ values by setting *y* = 50 and solving for *t*.

## Author contributions

Conceptualization of study (all authors); resources (MEE, RGE and others as specified in Acknowledgements); investigation and methodology (MEE, RGE and others as specified in Acknowledgements); formal analysis (MEE, PJH); visualization and writing of original draft (MEE, PJH); writing — review and editing (all authors); funding acquisition, project administration, and supervision (PJH).

## Data availability

The data supporting this article have been included as part of the ESI.†

## Conflicts of interest

PJH is an inventor on patents pertaining to Invader probes, which have been issued to the University Idaho.

## Supplementary Material

OB-023-D4OB01403K-s001
