# Supplementary material for: Recognition of mixed-sequence double-stranded DNA regions using chimeric Invader/LNA probes
Source: Org Biomol Chem. 2024 Oct 8;23(3):619–28. doi: 10.1039/d4ob01403k (PMC11482323; doi:10.1039/d4ob01403k)
Supplement: OB-023-D4OB01403K-s001 [file OB-023-D4OB01403K-s001.pdf]

Supplementary Information (SI) for Organic & Biomolecular Chemistry.
This journal is © The Royal Society of Chemistry 2024

Recognition of mixed-sequence double-stranded DNA regions using chimeric Invader/LNA probes

Michaela E. Everly,[a] Raymond G. Emehiser,[a] and Patrick J. Hrdlicka[a]

[a] Department of Chemistry, University of Idaho, Moscow, Idaho 83844-2343, USA

E-mail: hrdlicka@uidaho.edu

Electronic Supplementary Information

Definition of zipper nomenclature	S2
Additional discussion of the "nearest neighbor exclusion principle"	S2
ESI-MS spectra and HPLC chromatograms of **LNA1** and **LNA2** (Table S1 and Fig. S1)	S3
Representative thermal denaturation curves (Fig. S2)	S5
Additional discussion of thermal denaturation profiles of **LNA1** and **LNA2** (Fig. S3)	S7
Additional discussion concerning thermal denaturation profiles of chimeric probes (Fig. S4)	S9
UV-Vis characterization of the oligonucleotides studied herein (Fig. S5 and Table S2)	S12
Sequences and T_m values of DNA hairpins used in this study (Table S3)	S15
Representative electrophoretograms from dose-response experiments (Figs. S6–S8)	S16
Raw gel electrophoresis blots (Figs. S9–S32)	S18
Supplementary references	S30

Definition of zipper nomenclature. The relative arrangement between two 2′-*O*-(pyren-1-yl)methyl-RNA monomers on opposing strands in an Invader probe is described by the following nomenclature: the number *n* is the distance (measured in number of base-pairs) between the two monomers and has a positive value if a monomer is shifted toward the 5'-side of its strand relative to the monomer on the opposite strand, and has a negative value a monomer is shifted toward the 3'-side of its strand relative to the monomer on the opposite strand.

Additional discussion of the "nearest neighbor exclusion principle". According to the nearest neighbor exclusion principle, local intercalator densities exceeding one intercalator per two base-pairs are unfavorable in DNA duplexes due to limitations in local helix expandability (each intercalation event expands the duplex by ~3.4 Å), and because the stabilizing stacking interactions between neighboring base-pairs and a first intercalating moiety become perturbed upon intercalation of a second intercalator.[S1-S4] Double-stranded Invader probes, featuring two intercalators in the energetic hotspot between two base-pairs, therefore become partially unwound and labile.[S5,S6] Conversely, duplex formation between individual Invader strands and cDNA results in strongly stabilizing stacking interactions between the intercalator and flanking base-pairs (the nearest neighbor exclusion principle is no longer violated, as the local intercalator density is one intercalator per two base-pairs or less).

Table S1. ESI-MS data of LNAs used in this study[a]

ON	Sequence	**Calculated *m/z* (M-H)$^-$**	**Observed *m/z* (M-H)$^-$**
**LNA1**	5'-ggtatatataggC	4349.8	4350.4
**LNA2**	5'-gcctatatatacC	4271.8	4273.6

[a] LNA monomers are denoted in lower case letters ("c" = 5-methyl-cytosin-1-yl LNA monomer), C = cytosin-1-yl DNA monomer.

LNA1 **LNA2**

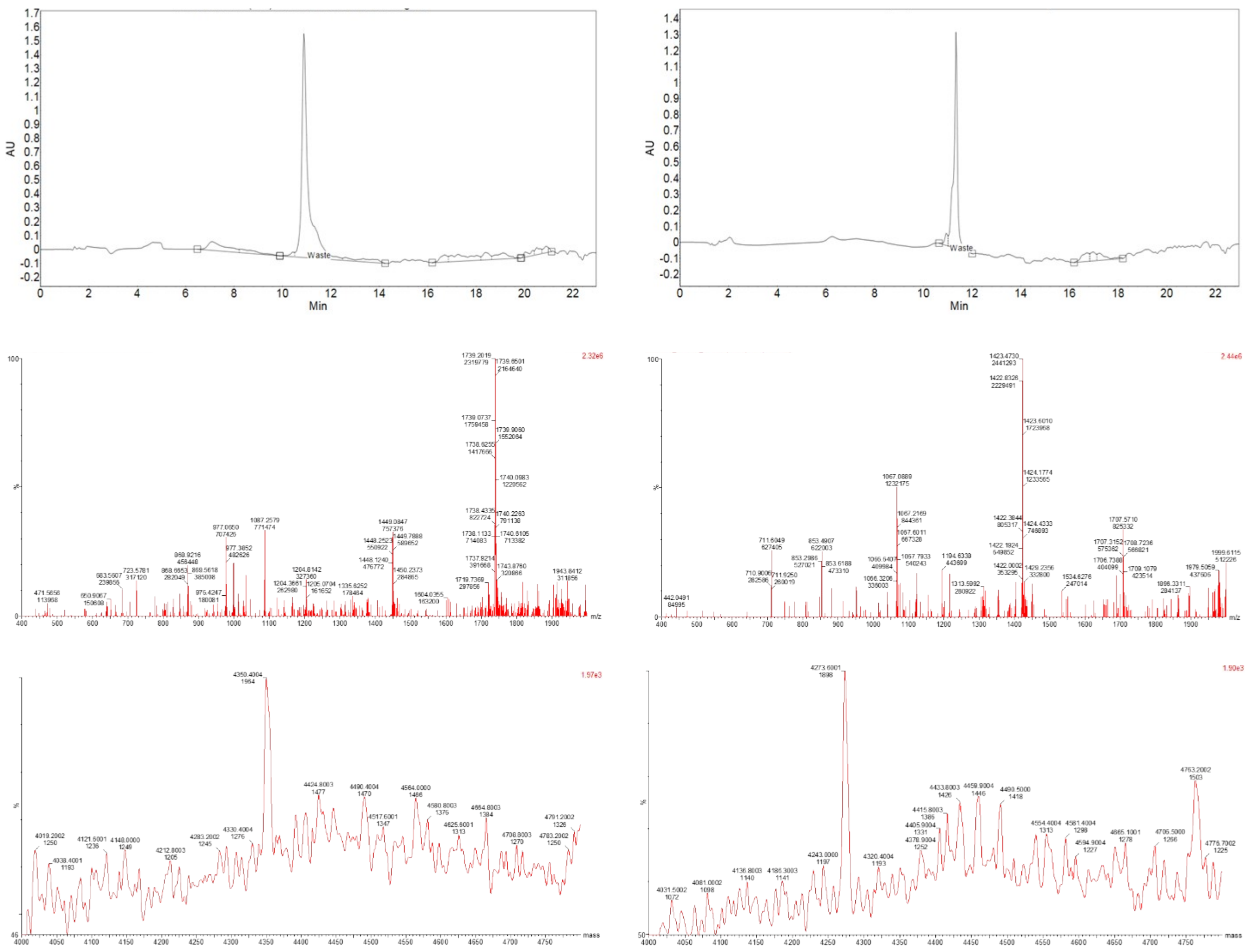

Figure S1. HPLC traces (upper panels), and unprocessed (middle panels) and deconvoluted (lower panels) ESI-MS spectra for **LNA1** and **LNA2**.

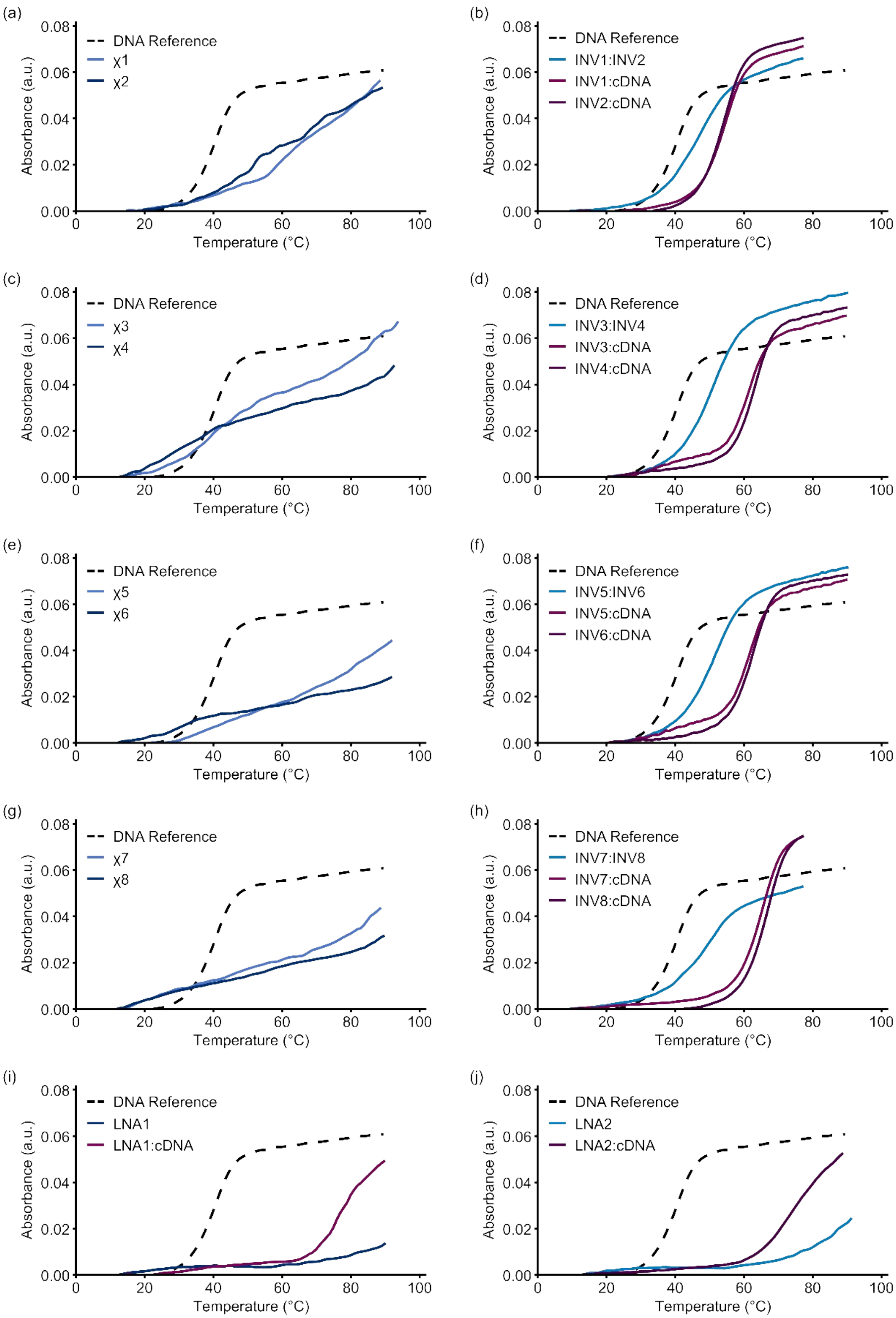

Figure S2. Representative thermal denaturation curves for chimeric Invader/LNA probes, Invader probes, Invader/cDNA, and LNA/cDNA duplexes. For sequences of strands, see Table 1. Chimeric

probes are comprised of the following strands: **χ1** (**INV1**:**LNA2**), **χ2** (**INV2**:**LNA1**), **χ3** (**INV3**:**LNA2**), **χ4** (**INV4**:**LNA1**), **χ5** (**INV5**:**LNA2**), **χ6** (**INV6**:**LNA1**), **χ7** (**INV7**:**LNA2**), and **χ8** (**INV8**:**LNA1**). For experimental conditions, see Table 1. Thermal denaturation curves for **INV3**:**INV4**, **INV5**:**INV6**, and **INV7**:**INV8** and the corresponding duplexes between individual Invader strands and cDNA were previously shown in references S7 and S8.

*Additional discussion of thermal denaturation profiles of **LNA1** and **LNA2**.* The thermal denaturation profiles of **LNA1** and **LNA2** appear to exhibit transitions at both low and very high temperatures (Fig. S2). Additional denaturation experiments were performed to further explore the potential formation and nature of secondary structures.

First, thermal denaturation profiles of **LNA1** and **LNA2** were recorded in low salt buffers to verify the high-melting transitions and rule out instrumental drift (Fig. S3a). Indeed, the high-melting transitions for **LNA1** and **LNA2** shifted to lower temperatures and became more distinct whereas the low-melting transitions were no longer observable (compare **LNA1** and **LNA2** profiles, Figs. S2 and S3a). This strongly suggests that the high-melting transitions are indeed due to denaturation of very stable secondary structures.

Next, denaturation profiles were recorded in which the concentrations of the LNA strands were increased 10-fold in both low and medium salt buffers (Figs. S3a and S3b). At low salt conditions, the high-melting transitions shifted to higher temperatures when the LNA strands were used at 10 μM concentration, suggesting that these transitions are due to denaturation of intermolecular structures (Fig. S3d). Conversely, the low-melting transitions were largely unaffected by the 10-fold increase in probe concentration (at medium salt conditions), indicating that these transitions are due to weak intramolecular interactions (Fig. S3c). Thus, **LNA1** and **LNA2** appear to form weakly associated hairpins and stable homo-dimers, i.e., secondary structures that should be expected to interfere with LNA-mediated dsDNA-recognition.

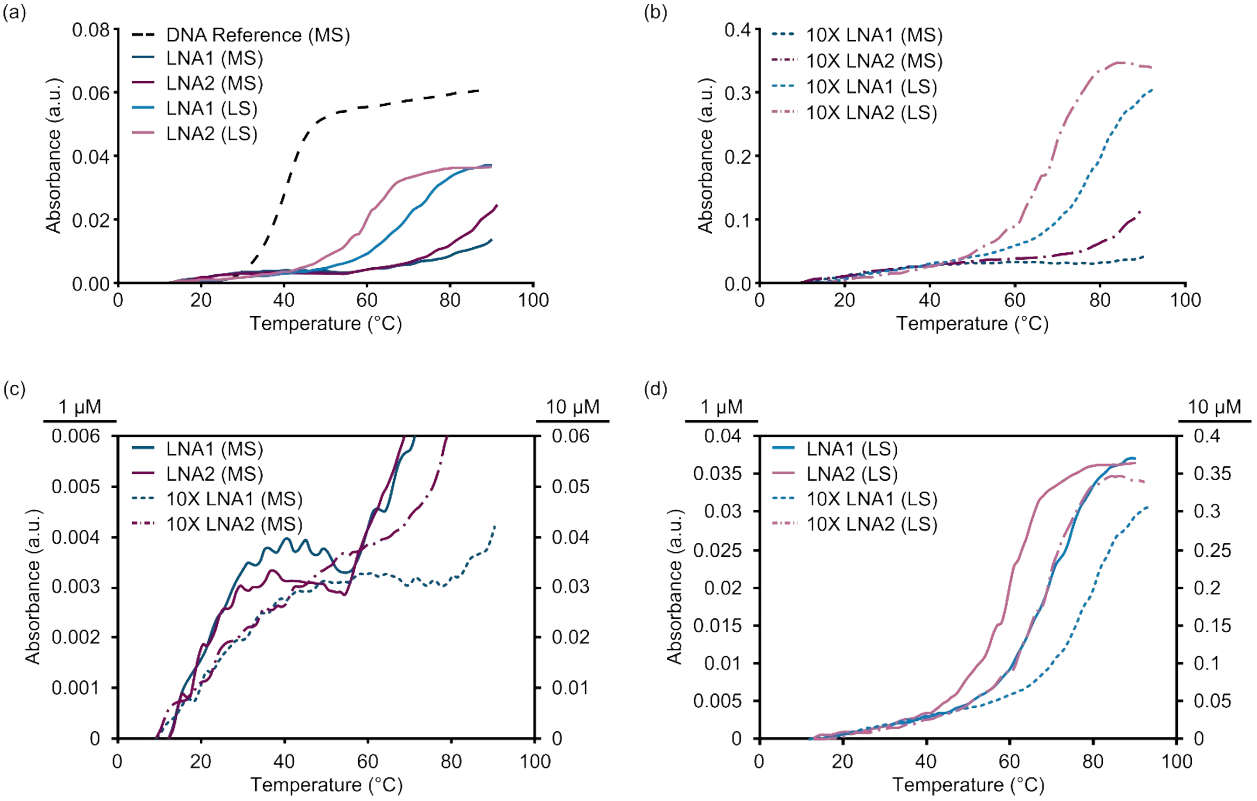

Figure S3. Representative thermal denaturation curves of **LNA1** and **LNA2** in medium salt (MS; $[Na^+]$ = 110 mM, $[Cl^-]$ = 100 mM, pH 7.0 (NaH_2PO_4/Na_2HPO_4), [EDTA] = 0.2 mM) or low salt (LS; $[Na^+]$ = 10 mM, pH 7.0 (NaH_2PO_4/Na_2HPO_4), [EDTA] = 0.2 mM) phosphate buffers using (a) 1.0 µM or (b) 10 µM of each strand. Panels (c) and (d) are zoom-in overlays of the low temperature (recorded in MS buffer) or high temperature transitions (recorded in LS buffer); left and right Y-axes depict A_{260} for probes used at 1 µM and 10 µM concentration, respectively. DNA Reference = corresponding unmodified DNA duplex (5′-GGTATATATAGGC:3′-CCATATATATCCG).

Additional discussion concerning thermal denaturation profiles of chimeric probes. To eliminate potential contributions of LNA-only secondary structures to the observed denaturation profiles of the chimeric Invader/LNA probes (Fig. S2), differential curves were constructed by subtracting LNA-only denaturation profiles from the corresponding chimeric probe profiles by using the Maths function in the Cary WinUV Thermal Application (Fig. S4). This allowed for a more reliable determination of T_m and TA values.

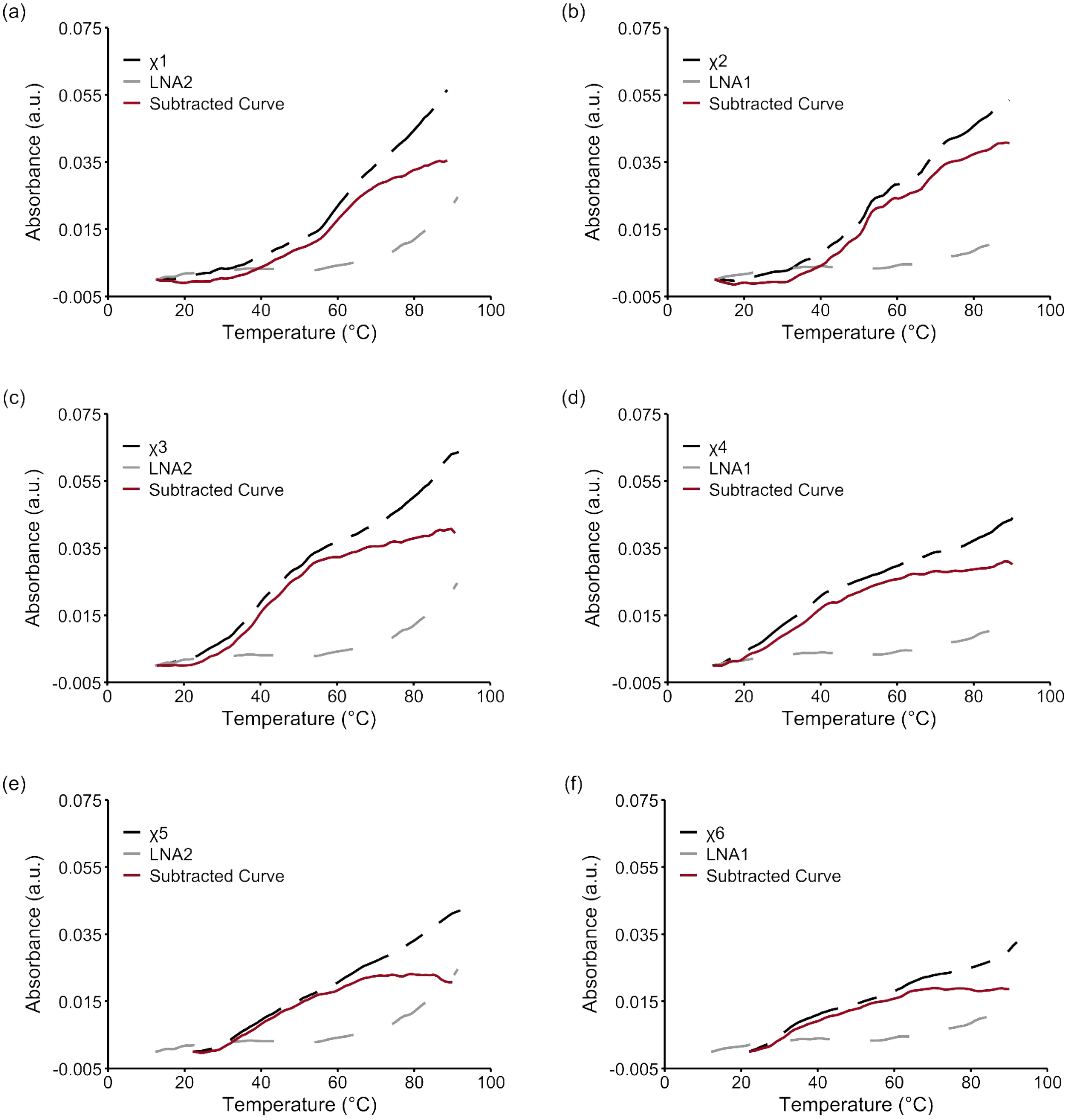
(a)
χ1
LNA2
Subtracted Curve
Absorbance (a.u.)
Temperature (°C)
(b)
χ2
LNA1
Subtracted Curve
Absorbance (a.u.)
Temperature (°C)
(c)
χ3
LNA2
Subtracted Curve
Absorbance (a.u.)
Temperature (°C)
(d)
χ4
LNA1
Subtracted Curve
Absorbance (a.u.)
Temperature (°C)
(e)
χ5
LNA2
Subtracted Curve
Absorbance (a.u.)
Temperature (°C)
(f)
χ6
LNA1
Subtracted Curve
Absorbance (a.u.)
Temperature (°C)

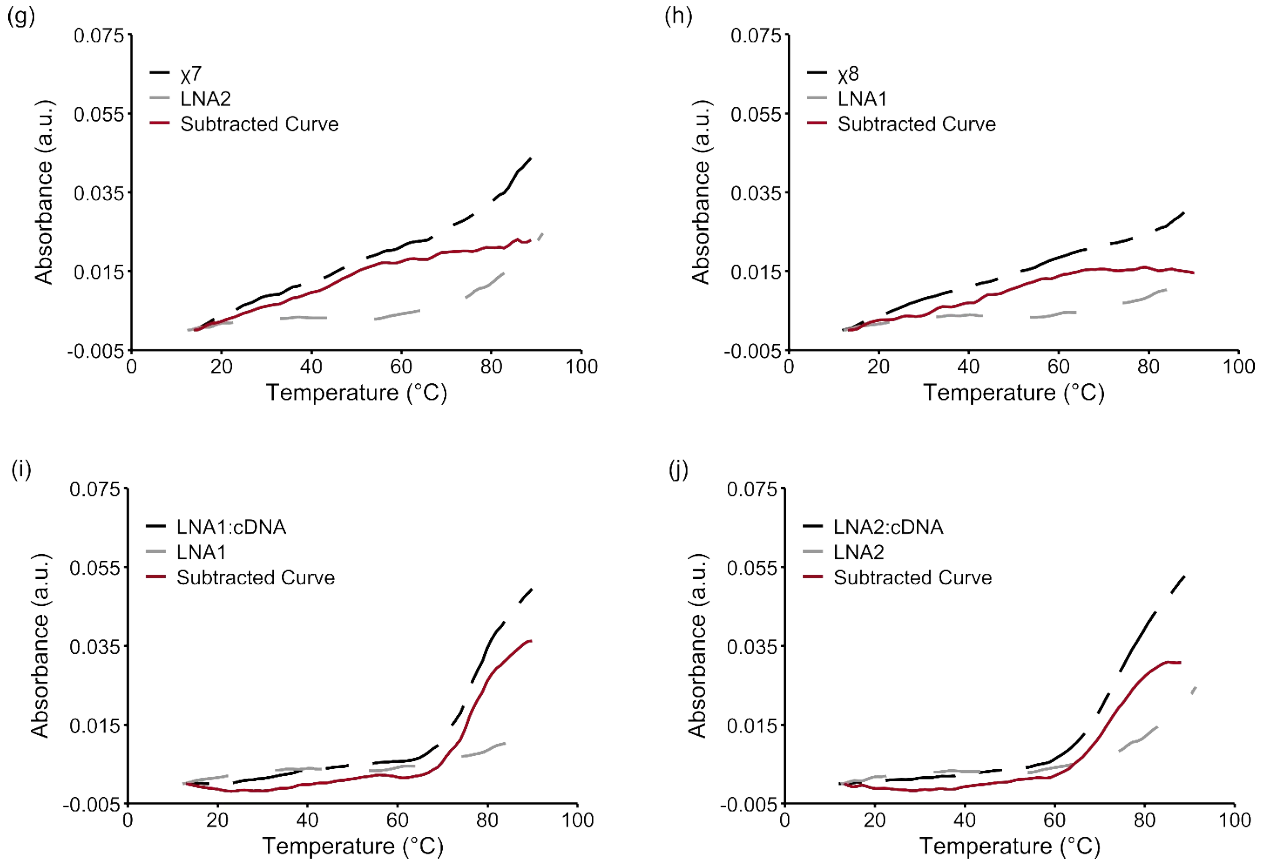

Figure S4. Representative differential thermal denaturation curves obtained by subtracting **LNA1** or **LNA2** profiles from the profiles of the corresponding double-stranded chimeric probe (a–h) or LNA/cDNA profiles (i, j). See Table 1 for experimental conditions.

UV-Vis characterization of the oligonucleotides studied herein. To gain insight into the placement of the pyrene moieties of the probes, UV-Vis spectra were recorded for individual Invader strands and the corresponding duplexes with complementary DNA, LNA, or Invader strands. Intercalation of the pyrene moieties upon duplex formation is expected to result in bathochromic shifts of the pyrene absorption bands relative to the individual Invader probe strands, whereas hypsochromic shifts are expected if duplex formation reduces electronic interactions between pyrene moieties and nucleobases.[S9]

Individual Invader strands display pyrene absorption maxima in the 333–334 nm and 348–350 nm range (Fig. S5, Table S2). Surprisingly, hybridization with cDNA only results in bathochromic shifts with the more highly modified Invader strands (Fig. S5, Table S2). While this contrasts our earlier findings,[S6,S10] this may reflect a certain level of structural pre-organization in the individual Invader strands. Formation of double-stranded Invader probes with three energetic hotspots results in significant hypsochromic shifts (Fig. S5, Table S2), consistent with perturbed pyrene-nucleobase stacking. No substantive changes in the location of absorption maxima were observed for chimeric Invader/LNA probes relative to single-stranded Invader strands (Fig. S5, Table S2). Thus, the data is inconclusive with respect to location of the pyrene moieties following chimeric probe assembly. However, it is evident from the thermal denaturation profiles that the chimeric probes adopt distorted duplex geometries.

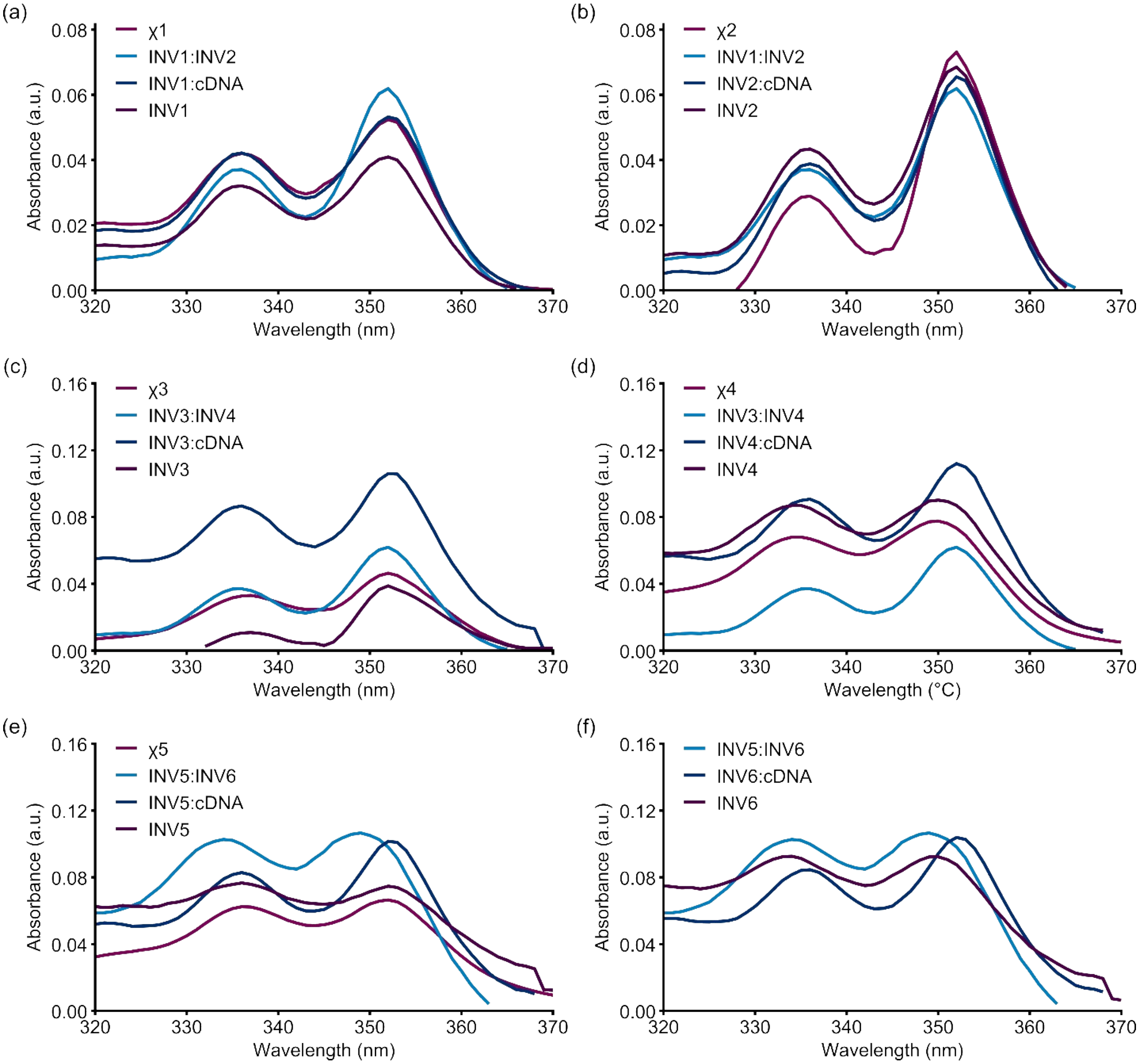

Figure S5. Representative UV-Vis absorption spectra for certain individual Invader strands and the corresponding duplexes with complementary DNA, LNA, and Invader strands. Spectra were recorded at 10 °C in medium salt buffer using quartz optical cells with a 1.0 cm path length. Some spectra shown here were previously reported.[S10]

Table S2. Absorption maxima in the 340–365 nm region for single-stranded Invader probes and the corresponding duplexes with complementary DNA, LNA, or Invader strands [a]

	λ_{max} (nm) [$\Delta\lambda_{max}$]			
**Probe**	**SSP**	**+INV**	**+cDNA**	**+LNA**
**INV1**	352	352 [±0]	352 [±0]	352 [±0]
**INV2**	352	352 [±0]	352 [±0]	352 [±0]
**INV3**	352	346 [−6]	352 [±0]	352 [±0]
**INV4**	349	346 [−3]	352 [+3]	350 [+1]
**INV5**	352	349 [−3]	352 [±0]	352 [±0]
**INV6**	350	349 [−1]	352 [+2]	n.d.
**INV7**	n.d.	n.d.	n.d.	n.d.
**INV8**	349	n.d.	351 [+2]	349 [±0]

[a] SSP = single-stranded probe. $\Delta\lambda_{max}$ is calculated relative to the single-stranded Invader strand. Binding partners (listed in the parenthesis) are as follows: **INV1** (**INV2** and **LNA2**), **INV2** (**INV1** and **LNA1**), **INV3** (**INV4** and **LNA2**), **INV4** (**INV3** and **LNA1**), **INV5** (**INV6** and **LNA2**), **INV6** (**INV5**), **INV7** (**INV8** and **LNA2**), and **INV8** (**INV7** and **LNA1**). Spectra were recorded at 10 °C in medium salt buffer using quartz optical cells with a 1.0 cm path length. n.d. = not determined due to limited probe quantity. Some data listed here were previously reported.[S10]

Table S3. Sequences and T_ms of DNA hairpins used in this study.[a]

Hairpin	Sequence	T_m (°C)
DH1	5'- G G T A T A T A T A G G C<br>3'- C C A T A T A T A T C C G<br>T_{10}	58.5
DH2	5'- G G T A T **T** T A T A G G C<br>3'- C C A T A **A** A T A T C C G<br>T_{10}	60.5
DH3	5'- G G T A T **G** T A T A G G C<br>3'- C C A T A **C** A T A T C C G<br>T_{10}	63.5
DH4	5'- G G T A T **C** T A T A G G C<br>3'- C C A T A **G** A T A T C C G<br>T_{10}	63.0
DH5	5'- G G T A T A T A **A** A G G C<br>3'- C C A T A T A T **T** T C C G<br>T_{10}	60.0
DH6	5'- G G T A T A T A **G** A G G C<br>3'- C C A T A T A T **C** T C C G<br>T_{10}	62.5
DH7	5'- G G T A T A T A **C** A G G C<br>3'- C C A T A T A T **G** T C C G<br>T_{10}	62.5

[a] For experimental conditions, see Table 1. T_m values have been previously reported in reference S8 but are included here for convenience.

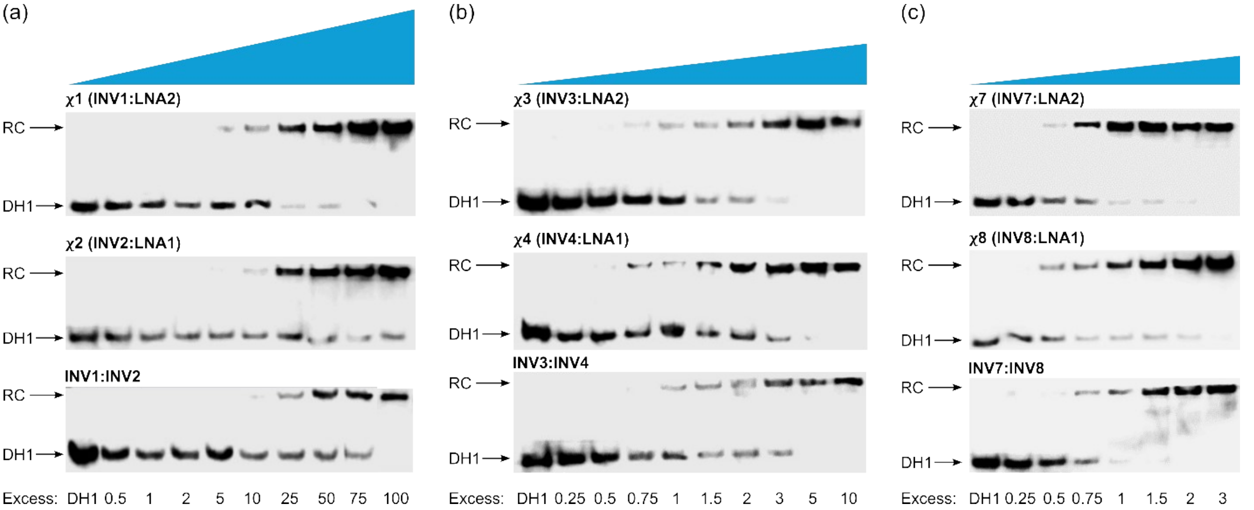

Figure S6. Representative electrophoretograms for dose-response experiments in which **DH1** (50 nM) was incubated alone (left lanes) or with variable molar fold-excess of (a) 2X-, (b) 3X-, or (c) 4X-modified chimeric and Invader probes at 37 °C for ~17 h. RC = recognition complex. Conditions are otherwise as described in Fig. 3. The electrophoretogram for **INV1**:**INV2** is a composite of two separate gels.

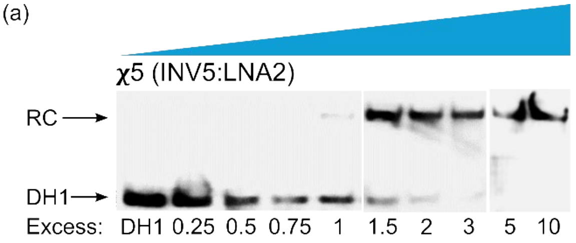

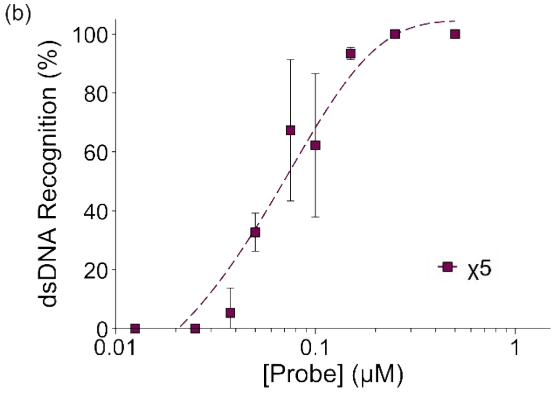

(c)

Probe	C_{50} (nM)	Rec_{5X} (%)
χ5	69.5	100 ± 0

Figure S7. (a) Representative electrophoretogram and (b) dose-response curve in which **DH1** (50 nM) was incubated alone (left lane) or with variable molar fold-excess of **χ5**. Dose-response curve was used to calculate the corresponding (c) C_{50} value. RC = recognition complex. The electrophoretogram is a composite of two separate gels. Conditions are as described in Fig. 3.

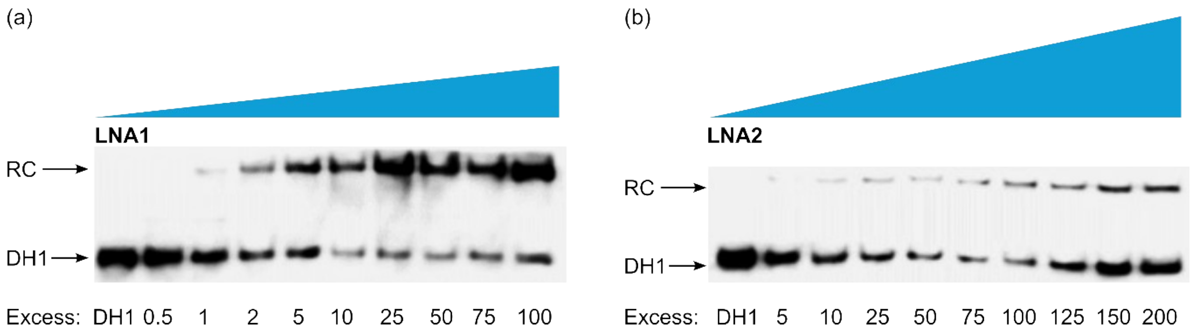

Figure S8. Representative electrophoretograms for dose-response experiments in which **DH1** (50 nM) was incubated alone (left lands) or with variable molar fold-excess of (a) **LNA1** or (b) **LNA2**. RC = recognition complex. Conditions are as described in Fig. 3.

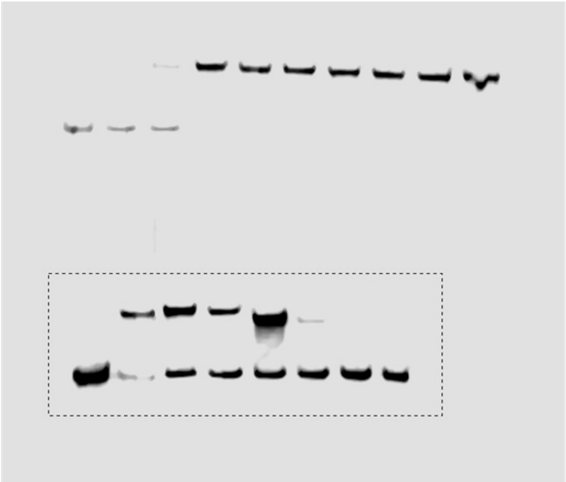

Figure S9. Uncropped image of gel blot for electrophoretogram in Fig. 3a. The full image was processed using Image Studio Digits Ver 5.2 software as follows: first, the image was subjected to the “Noise Removal” operation (1x). Second, contrast was adjusted such that background signals were reduced but all bands remained visible. Dashed box indicates the specified electrophoretogram. All bands located above the dashed box are from a separate gel blot that was simultaneously imaged with the specified blot.

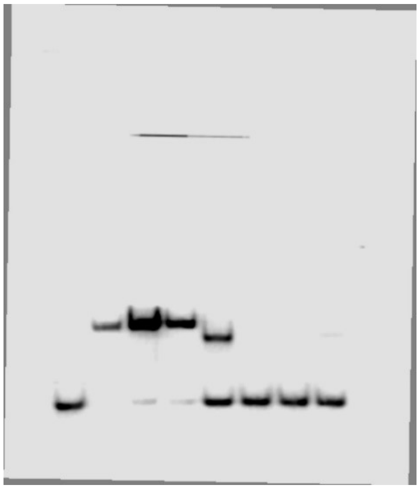

Figure S10. Uncropped image of gel blot for electrophoretogram in Fig. 3b. Image was processed as described in Fig. S9. The horizontal line above the electrophoretogram is from the surface of the scanner and not the blot.

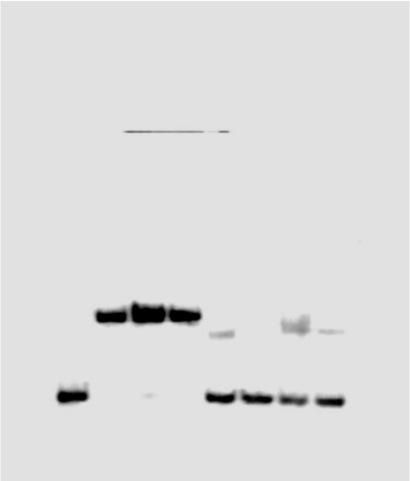

Figure S11. Uncropped image of gel blot for electrophoretogram in Fig. 3c. Image was processed as described in Fig. S9. The horizontal line above the electrophoretogram is from the surface of the scanner and not the blot.

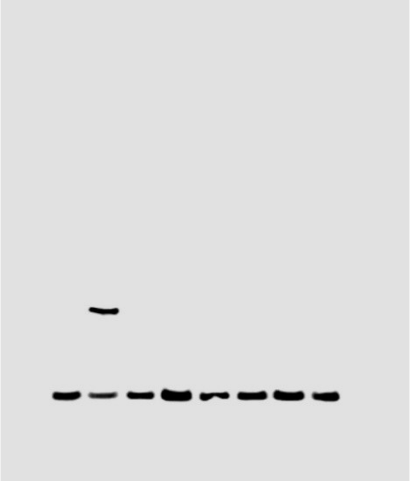

Figure S12. Uncropped image of gel blot for electrophoretogram of **χ2** (25-fold) in Fig. 5 (middle panel). Image was processed as described in Fig. S9. Leftmost band corresponds to **DH1** incubated alone as a reference and was excluded from cropped image in the main manuscript.

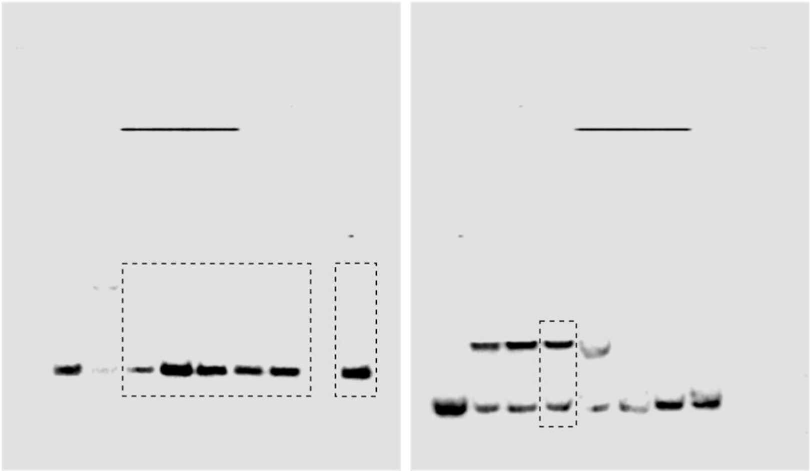

Figure S13. Uncropped images of gel blots for electrophoretogram composite of **INV1**:**INV2** (25-fold) in Fig. 5 (middle panel). Image was processed as described in Fig. S9. The horizontal line above both electrophoretograms is from the surface of the scanner and not the blots. Dashed boxes indicate the lanes used for composite.

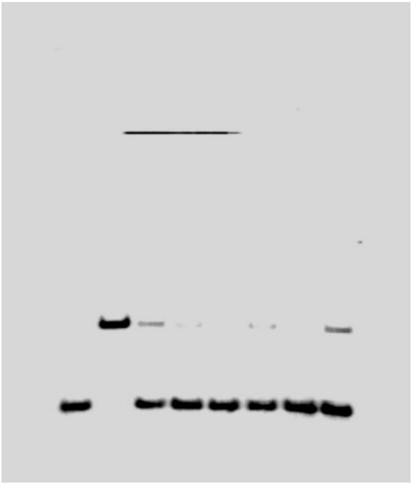

Figure S14. Uncropped image of gel blot for electrophoretogram of **χ4** (25-fold) in Fig. 5 (middle panel). Image was processed as described in Fig. S9. Leftmost band corresponds to **DH1** incubated alone as a reference and was excluded from cropped image in the main manuscript. The horizontal line above the electrophoretogram is from the surface of the scanner and not the blot.

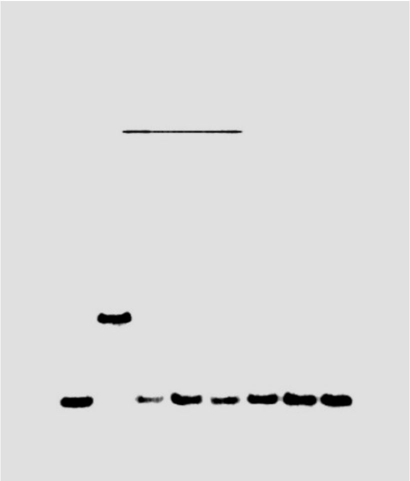

Figure S15. Uncropped image of gel blot for electrophoretogram of **INV3**:**INV4** (25-fold) in Fig. 5 (middle panel). Image was processed as described in Fig. S9. Leftmost band corresponds to **DH1** incubated alone as a reference and was excluded from cropped image in the main manuscript. The horizontal line above the electrophoretogram is from the surface of the scanner and not the blot.

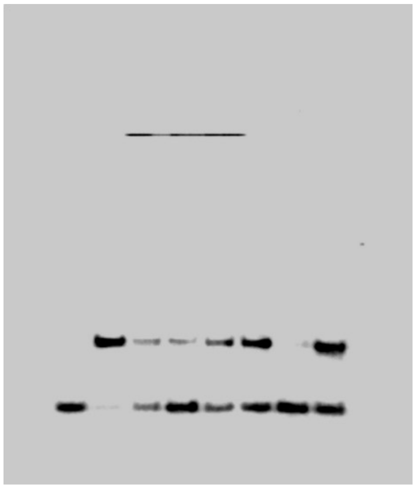

Figure S16. Uncropped image of gel blot for electrophoretogram of **LNA1** (25-fold) in Fig. 5 (middle panel). Image was processed as described in Fig. S9. Leftmost band corresponds to **DH1** incubated alone as a reference and was excluded from cropped image in the main manuscript. The horizontal line above the electrophoretogram is from the surface of the scanner and not the blot.

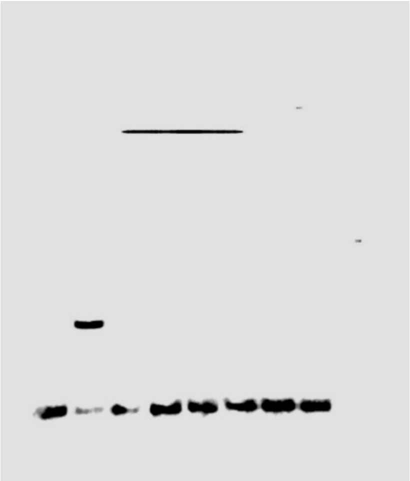

Figure S17. Uncropped image of gel blot for electrophoretogram of **χ4** (3-fold) in Fig. 5 (lower panel). Image was processed as described in Fig. S9. Leftmost band corresponds to **DH1** incubated alone as a reference and was excluded from cropped image in the main manuscript. The horizontal line above the electrophoretogram is from the surface of the scanner and not the blot.

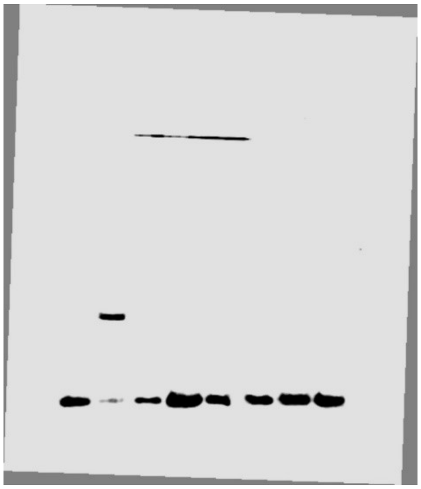

Figure S18. Uncropped image of gel blot for electrophoretogram of **INV3**:**INV4** (3-fold) in Fig. 5 (lower panel). Image was processed as described in Fig. S9. Leftmost band corresponds to **DH1** incubated alone as a reference and was excluded from cropped image in the main manuscript. The horizontal line above the electrophoretogram is from the surface of the scanner and not the blot.

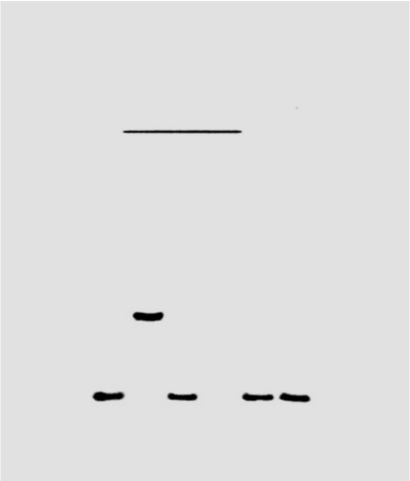

Figure S19. Uncropped image of gel blot for electrophoretogram of **χ8** (3-fold) in Fig. 5 (lower panel). Image was processed as described in Fig. S9. Leftmost band, corresponding to **DH1** incubated alone as a reference, and the empty lane were excluded from cropped image in the main manuscript. The horizontal line above the electrophoretogram is from the surface of the scanner and not the blot.

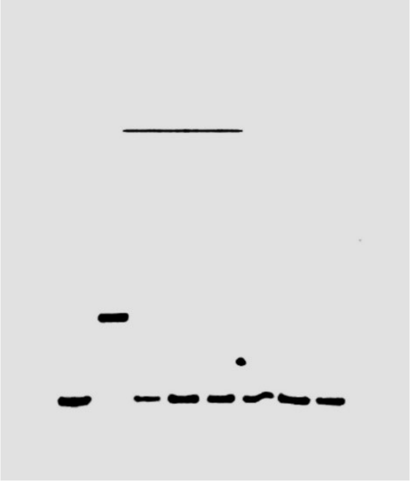

Figure S20. Uncropped image of gel blot for electrophoretogram of **INV7**:**INV8** (3-fold) in Fig. 5 (lower panel). Image was processed as described in Fig. S9. Leftmost band corresponds to **DH1** incubated alone as a reference and was excluded from cropped image in the main manuscript. The horizontal line above the electrophoretogram is from the surface of the scanner and not the blot.

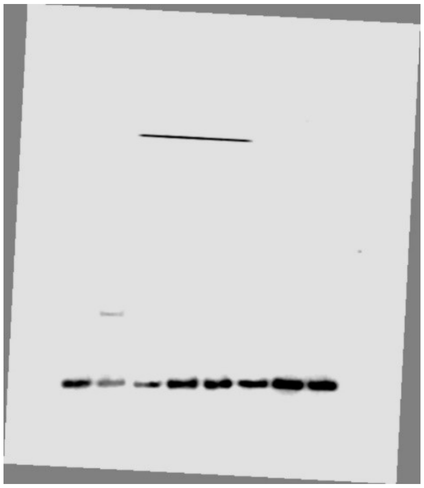

Figure S21. Uncropped image of gel blot for electrophoretogram of **LNA1** (3-fold) in Fig. 5 (lower panel). Image was processed as described in Fig. S9. Leftmost band corresponds to **DH1** incubated alone as a reference and was excluded from cropped image in the main manuscript. The horizontal line above the electrophoretogram is from the surface of the scanner and not the blot.

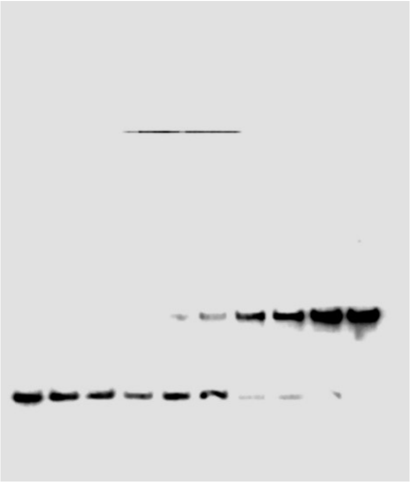

Figure S22. Uncropped image of gel blot for electrophoretogram of **χ1** dose-response in Fig. S6a. Image was processed as described in Fig. S9. The horizontal line above the electrophoretogram is from the surface of the scanner and not the blot.

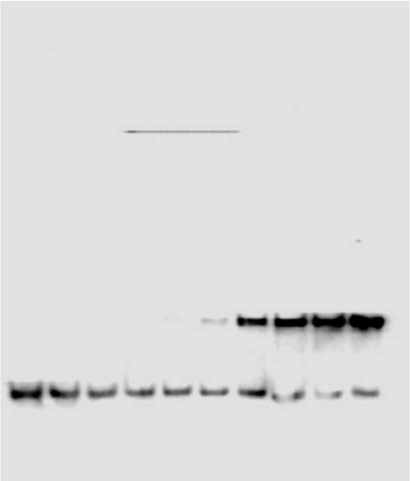

Figure S23. Uncropped image of gel blot for electrophoretogram of **χ2** dose-response in Fig. S6a. Image was processed as described in Fig. S9. The horizontal line above the electrophoretogram is from the surface of the scanner and not the blot.

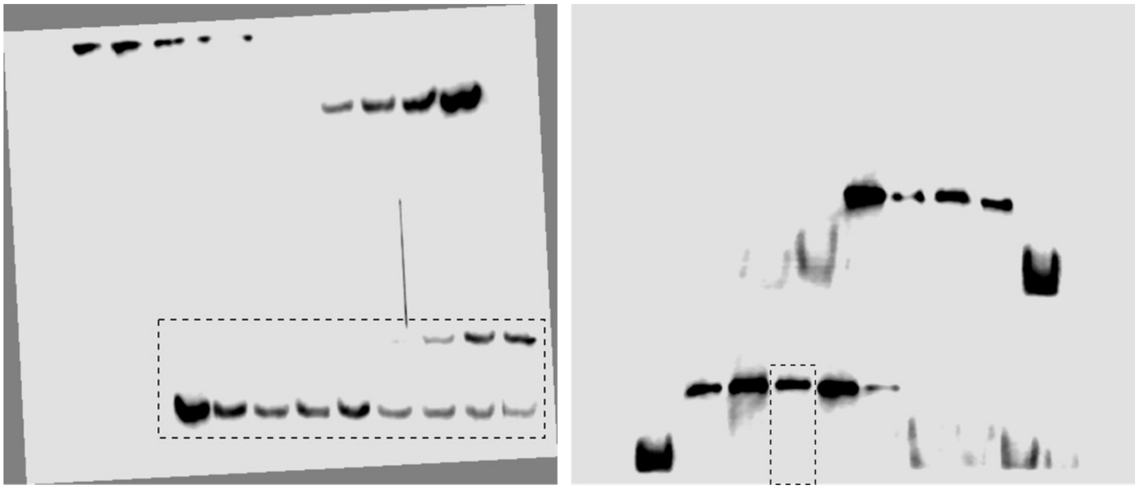

Figure S24. Uncropped images of gel blots for electrophoretogram composite of **INV1**:**INV2** dose-response in Fig. S6a. Image was processed as described in Fig. S9. The vertical line in left image is from the surface of the scanner and not the blot. Dashed boxes indicate the lanes used for composite. All bands located above the dashed boxes are from separate gel blots that were simultaneously imaged with the specified blot.

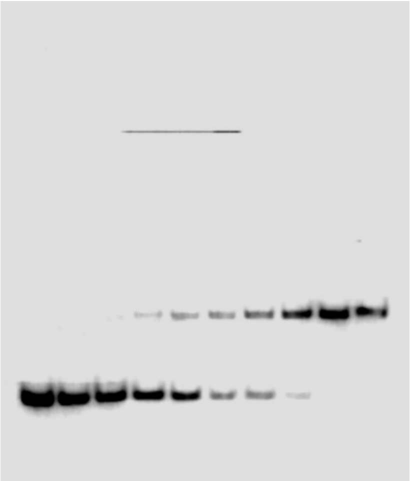

Figure S25. Uncropped image of gel blot for electrophoretogram of **χ3** dose-response in Fig. S6b. Image was processed as described in Fig. S9. The horizontal line above the electrophoretogram is from the surface of the scanner and not the blot.

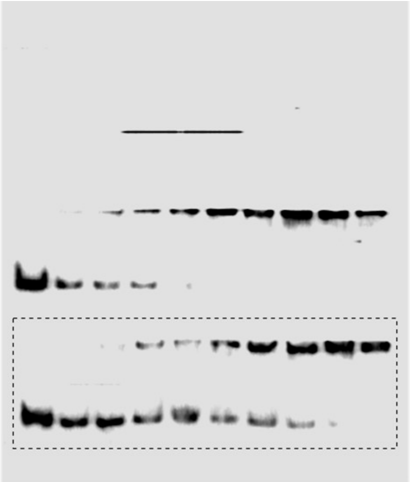

Figure S26. Uncropped image of gel blot for electrophoretogram of **χ4** dose-response in Fig. S6b. Image was processed as described in Fig. S9. The horizontal line above the electrophoretogram is from the surface of the scanner and not the blot. Dashed box indicates the specified electrophoretogram.

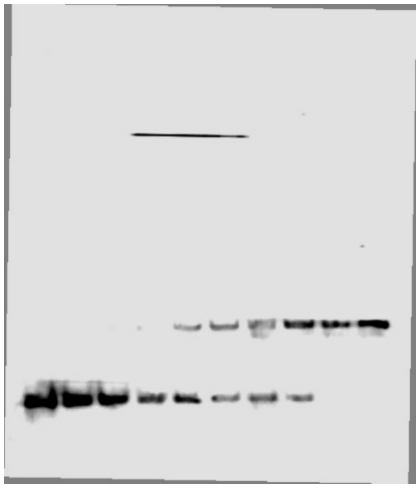

Figure S27. Uncropped image of gel blot for electrophoretogram of **INV3**:**INV4** dose-response in Fig. S6b. Image was processed as described in Fig. S9. The horizontal line above the electrophoretogram is from the surface of the scanner and not the blot.

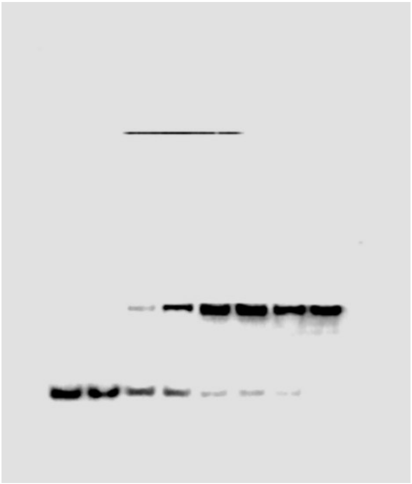

Figure S28. Uncropped image of gel blot for electrophoretogram of **χ7** dose-response in Fig. S6c. Image was processed as described in Fig. S9. The horizontal line above the electrophoretogram is from the surface of the scanner and not the blot.

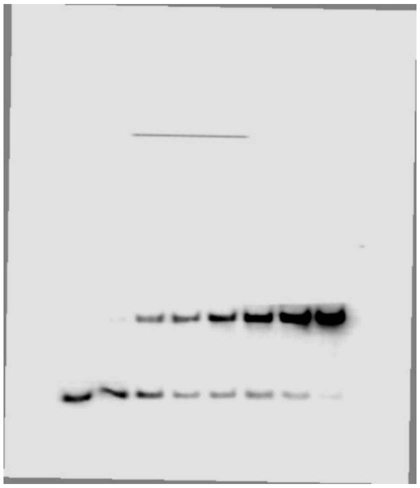

Figure S29. Uncropped image of gel blot for electrophoretogram of χ**8** dose-response in Fig. S6c. Image was processed as described in Fig. S9. The horizontal line above the electrophoretogram is from the surface of the scanner and not the blot.

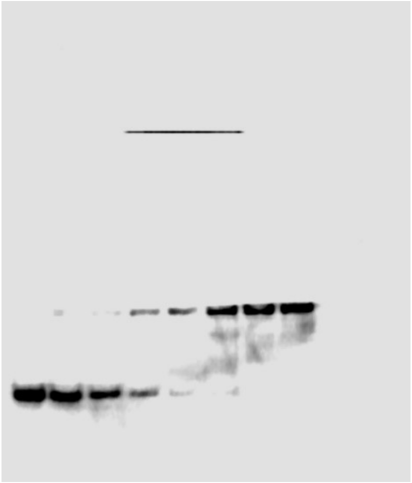

Figure S30. Uncropped image of gel blot for electrophoretogram of **INV7**:**INV8** dose-response in Fig. S6c. Image was processed as described in Fig. S9. The horizontal line above the electrophoretogram is from the surface of the scanner and not the blot.

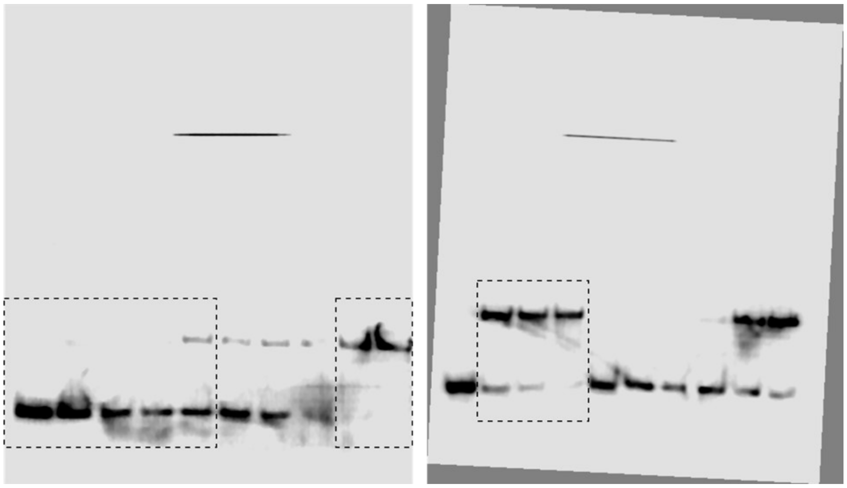

Figure S31. Uncropped images of gel blots for electrophoretogram composite of **χ5** dose-response in Fig. S7a. Image was processed as described in Fig. S9. The horizontal line above the electrophoretogram is from the surface of the scanner and not the blot. Dashed boxes indicate the lanes used for composite.

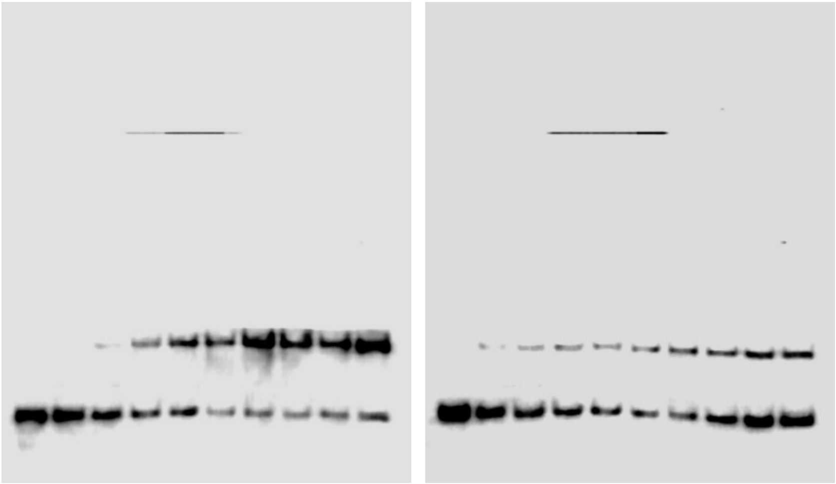

Figure S32. Uncropped images of gel blots for electrophoretograms of **LNA1** (left) and **LNA2** (right) dose-response in Figs. S8a and S8b. Images were processed as described in Fig. S9. The horizontal line above both electrophoretograms is from the surface of the scanner and not the blots.

SUPPLEMENTARY REFERENCES

S1) H. Ihmels and D. Otto, *Top. Curr. Chem.* 2005, **258**, 161–204.

S2) O. Persil and N. V. Hud, *Trends Biotechnol.* 2007, **25**, 433–436.

S3) S. C. Jain, C. Tsai and H. M. Sobell, *J. Mol. Biol.* 1977, **114**, 317–331.

S4) L. D. Williams, M. Egli, Q. Gao and A. Rich, A. DNA intercalation: Helix unwinding and neighbor-exclusion. In Structure and Function: Nucleic Acids; R. H. Sarma and M. H. Sarma, Eds.; Adenine Press: Albany, NY, USA, 1992; Volume 1, pp. 107–125.

S5) S. P. Sau, A. S. Madsen, P. Podbevsek, N. K. Andersen, T. S. Kumar, S. Andersen, R. L. Rathje, B. A. Anderson, D. C. Guenther, S. Karmakar, P. Kumar, J. Plavec, J. Wengel and P. J. Hrdlicka, *J. Org. Chem.* 2013, **78**, 9560–9570.

S6) S. Karmakar, A. S. Madsen, D. C. Guenther, B. C. Gibbons and P. J. Hrdlicka, *Org. Biomol. Chem.*, 2014, **12**, 7758–7773.

S7) R. Emehiser, E. Hall, D. C. Guenther, S. Karmakar and P. J. Hrdlicka, *Org. Biomol. Chem.*, 2020, **18**, 56–65.

S8) D. C. Guenther, G. H. Anderson, S. Karmakar, B. A. Anderson, B. A. Didion, W. Guo, J. P. Verstegen and P. J. Hrdlicka, *Chem. Sci.*, 2015, **6**, 5006–5015.

S9) H. Asanuma, T. Fujii, T. Kato and H. Kashida, *J. Photochem. Photobiol., C*, 2012, **13**, 124–135.

S10) R. Emehiser and P. J. Hrdlicka, *Org. Biomol. Chem.*, 2020, **18**, 1359–1369.
